# Differential Early *in vivo* Dynamics and Functionality of Recruited Polymorphonuclear Neutrophils After Infection by Planktonic or Biofilm *Staphylococcus aureus*

**DOI:** 10.3389/fmicb.2021.728429

**Published:** 2021-08-30

**Authors:** Aizat Iman Abdul Hamid, Andréa Cara, Alan Diot, Frédéric Laurent, Jérôme Josse, Pascale Gueirard

**Affiliations:** ^1^Laboratoire Microorganismes: Génome et Environnement, CNRS UMR 6023, Université Clermont Auvergne, Clermont-Ferrand, France; ^2^Centre International de Recherche et Infectiologie, Inserm U1111, CNRS UMR 5308, École Normale Supérieure de Lyon, Université Claude Bernard Lyon 1, Lyon, France

**Keywords:** *Staphylococcus aureus*, biofilm, innate immunity, polymorphonuclear neutrophils, macrophages, murine model, intravital imaging

## Abstract

*Staphylococcus aureus* is a human pathogen known for its capacity to shift between the planktonic and biofilm lifestyles. *In vivo*, the antimicrobial immune response is characterized by the recruitment of inflammatory phagocytes, namely polymorphonuclear neutrophils (PMNs) and monocytes/macrophages. Immune responses to planktonic bacteria have been extensively studied, but many questions remain about how biofilms can modulate inflammatory responses and cause recurrent infections in live vertebrates. Thus, the use of biologically sound experimental models is essential to study the specific immune signatures elicited by biofilms. Here, a mouse ear pinna model of infection was used to compare early innate immune responses toward *S. aureus* planktonic or biofilm bacteria. Flow cytometry and cytokine assays were carried out to study the inflammatory responses in infected tissues. These data were complemented with intravital confocal imaging analyses, allowing the real-time observation of the dynamic interactions between EGFP + phagocytes and bacteria in the ear pinna tissue of LysM-EGFP transgenic mice. Both bacterial forms induced an early and considerable recruitment of phagocytes in the ear tissue, associated with a predominantly pro-inflammatory cytokine profile. The inflammatory response was mostly composed of PMNs in the skin and the auricular lymph node. However, the kinetics of PMN recruitment were different between the 2 forms in the first 2 days post-infection (pi). Two hours pi, biofilm inocula recruited more PMNs than planktonic bacteria, but with decreased motility parameters and capacity to emit pseudopods. Inversely, biofilm inocula recruited less PMNs 2 days pi, but with an “over-activated” status, illustrated by an increased phagocytic activity, CD11b level of expression and ROS production. Thus, the mouse ear pinna model allowed us to reveal specific differences in the dynamics of recruitment and functional properties of phagocytes against biofilms. These differences would influence the specific adaptive immune responses to biofilms elicited in the lymphoid tissues.

## Introduction

*Staphylococcus aureus* (*S. aureus*) is a Gram-positive commensal bacterium that is also a leading cause of various invasive infections from soft skin tissue colonization to infections on implanted medical devices such as prosthetic joints. The wide range of staphylococcal virulence factors coupled with the apparition of community and hospital associated methicillin-resistant *S. aureus* (MRSA) strains have made infections by this bacterial species a particularly difficult therapeutic challenge (Turner et al., 2019; de Vor et al., 2020; Pidwill et al., 2021). Furthermore, the intrinsic capacity of *S. aureus* to adapt to its environment contributes toward its survival inside host tissues. An example of this type of adaptation is the capacity to form biofilms which are, contrary to the free-floating planktonic form of growth, microbial communities encased in a self-produced matrix composed of extracellular DNA (eDNA), proteins and polysaccharides (Moormeier and Bayles, 2017). According to the US Center for Disease Control, approximately 65% of human infections involve biofilms, with *S. aureus* accounting for up to 50% of pathogens isolated from prosthetic joint infections (Costerton, 2001; Ricciardi et al., 2018). Moreover, biofilms are often implicated in recurrent infections due to their increased tolerance toward antimicrobial treatments and even host immune attacks (Bjarnsholt, 2013).

Typically, polymorphonuclear neutrophils (PMNs) and monocytes/macrophages (MOs/MΦs) are rapidly recruited to infected tissue by microbe derived signals like formylated peptides or chemokines such as chemokine ligand 1 (CXCL1) and monocyte chemotactic protein-1 (MCP-1) (Capucetti et al., 2020; de Vor et al., 2020; Pidwill et al., 2021). These innate immune cells subsequently realize phagocytosis and efficiently kill planktonic bacteria through various effector mechanisms, including acidification of phagolysosomes and release of neutrophil extracellular traps (NETs) (de Vor et al., 2020; Pidwill et al., 2021). However, the transition to the biofilm lifestyle not only modifies the spatial distribution of *S. aureus* in colonized tissues, but is also accompanied by a shift in virulence mechanisms, both factors contributing toward the immunosuppressive nature of biofilms and thus to a recalcitrance toward elimination by innate immune phagocytes (Horn and Kielian, 2020; de Vor et al., 2020; Pidwill et al., 2021). *In vitro*, MΦs at the surface of *S. aureus* biofilms were mostly viable and retained a round morphology. However, the majority of cells that successfully penetrated biofilms were not viable, illustrating the protective barrier role that biofilms play in immune evasion (Thurlow et al., 2011; Gries et al., 2020a). Recently, most of the insights into the immune evasion mechanisms elicited by staphylococcal biofilms have been gained from *in vivo* models of skin/abscess infection or from biofilm infections on implanted medical devices (Horn and Kielian, 2020; Pidwill et al., 2021). These models have highlighted anti-inflammatory responses elicited by *S. aureus* biofilms due in part to the polarization of recruited MΦs toward an M2 anti-inflammatory phenotype, and also to the recruitment of an immature population of myeloid cells known as myeloid-derived suppressor cells (MDSCs) which were present as early as day 3 in infected knee joint tissue (Thurlow et al., 2011; Heim et al., 2018). Inversely, adoptive transfer of M1 MΦs, preactivated by IFN-γ or TNF-α and *S. aureus*–derived peptidoglycan, to the infectious tissue surrounding a biofilm inoculated catheter ameliorated immune responses and biofilm clearance (Hanke et al., 2013).

Even though PMNs are key cells in the *S. aureus* innate immune responses, little is known about the specific *in vivo* interactions between PMNs and biofilms. Indeed, while many *in vitro* studies have analyzed the effects of the various virulence factors of *S. aureus* biofilms, from the expression of PMN-killing toxins to NET degradation (de Vor et al., 2020), most *in vivo* knowledge has been restricted to FACS analyses of PMN recruitment toward infected tissue. Furthermore, most models studying innate immune responses toward *S. aureus* biofilms do not include comparisons with planktonic bacteria.

Thus, the early inflammatory responses toward both forms of *S. aureus* were characterized in a previously developed mouse soft skin tissue infection model. In these works, either *S. aureus* planktonic or biofilm bacteria were intradermally inoculated into the mouse ear pinna, which allowed the study of their effects on immune cell dynamics using intravital confocal microscopy (Forestier et al., 2017; Abdul Hamid et al., 2020; Sauvat et al., 2020). The use of this type of imaging in the context of biofilm infections has rarely been done but has allowed new insights into the study of immune responses from a cellular dynamic perspective (Gries et al., 2020b). Here, classic immunology techniques were coupled with intravital confocal imaging to rigorously compare inflammatory responses and phagocyte behavior in response to either the planktonic or biofilm form of *S. aureus*. Results show that early immune responses were globally similar at the tissue and cytokine levels but were significantly changed when analyzing cellular dynamics and specific interactions with bacteria. These data were coherent with modifications in the functional properties of phagocytes recruited toward the cutaneous infection site.

## Materials and Methods

### Mice and Ethics Statement

C57BL/6J WT mice (6–16-week-old males and females) were purchased from Charles River Laboratories. LysM-EGFP transgenic mice (6–16-week-old males and females) were obtained from the bacteria-cell interactions unit of the Pasteur Institute (Paris, France). Mice were bred in the animal care facility at Clermont Auvergne University (Clermont-Ferrand, France). All experiments were approved by the local Ethics Committee on Animal Experimentation (Auvergne C2E2A, Clermont-Ferrand, France, agreement number: 1,725) and were carried out in accordance with the applicable guidelines and regulations. All mice were provided an appropriate environment including shelter in cages, a comfortable resting area, sufficient space (not more than 5 animals per cage) and ready access to fresh water and food to maintain full health and vigor. Animal welfare was observed daily to ensure optimal conditions and treatment which avoid suffering. Littermates destined to be inoculated were housed in separate cages with access to the same facilities previously stated. The anesthetic used during experiments was chosen to promote deep anesthesia. During and after anesthesia, mice were kept warm in order to prevent any risks related to hypothermia. Euthanasia was performed by cervical dislocation on the anesthetized animal at the end of the infection period.

### SH1000 mCherry and GFP-Tagged *Staphylococcus aureus* Strain Construction

The *S. aureus* SH1000 strain was chosen for its capacity to produce high quantities of biofilm *in vitro* (Tasse et al., 2018), and used in all the experiments presented in this work. As described previously, the SH1000 strain possess a functional *agr* system (Herbert et al., 2010). The *S. aureus* SH1000 GFP-tagged fluorescent strain (named GFP-SH1000) was constructed after insertion of the pCN47-GFP plasmid (Charpentier et al., 2004) by electroporation into the SH1000 strain (O’Neill, 2010), as described previously (Schenk and Laddaga, 1992). The GFP-SH1000 strain was then selected onto Luria-Bertani (LB) agar containing erythromycin (10 μg/mL). Clones were grown overnight with shaking in Trypticase Soy broth (TSB) containing erythromycin (10 μg/mL) and stored at –80°C in the same medium with 15% glycerol. Fluorescence was detected in bacterial suspensions by fluorescence microscopy. The *S. aureus* SH1000 mCherry-tagged fluorescent strain (named mCherry-SH1000) was constructed in the same way with the pCN47-mCherry plasmid.

### Reagents and Monoclonal Antibodies

The reagents used in this study were as follows: collagenase, Type IV (Gibco), Deoxyribonuclease I from bovine pancreas (Sigma-Aldrich), EDTA (Invitrogen), Erythromycin (Sigma-Aldrich), Gentamicin (Sigma-Aldrich), LB agar (Condalab), Liberase^TM^ Research Grade (Roche), Pierce protease inhibitor (Thermo Fisher Scientific), PBS (Dutscher), RPMI 1640 w/stable glutamine (Dutscher), and TSB (BD Bacto). Antibodies and dyes from Miltenyi Biotec include: anti-mouse CD3ε (clone 17A2), CD335 (clone REA815), CD19 (clone REA749), CD45 (clone REA737), CD11b (clone REA592), Ly6G (clone REA526), Ly6C (clone REA796), Viobility 405/452 fixable dye, REA Control-APC (clone REA 293), REA Control-FITC (clone REA 293), REA Control-PE-Vio 770 (clone REA 293), REA Control- APC-Vio 770 (clone REA 293), REA Control- APC-VioBlue (clone REA 293), and REA Control- APC-VioGreen (clone REA 293).

### Bacterial Growth Conditions and Inocula Preparation

WT (non-fluorescent), GFP and mCherry-SH1000 strains were used in all the experiments performed. WT planktonic bacterial cultures were prepared from an aliquot of frozen bacteria in 5 mL of TSB and placed overnight at 37°C with agitation under aerobic conditions. Culture media of fluorescent strains were supplemented with erythromycin (10 μg/mL).

Planktonic inocula were prepared by first homogenizing overnight cultures and then determining bacterial concentration (CFU/mL), deduced by measuring the optical density at 600 nm (OD_60__0__*n*__*m*_) and multiplying it by the known titer (CFU/OD_60__0__*n*__*m*_) of the strain. The corresponding volume of overnight culture containing 5 × 10^6^ CFUs was withdrawn and centrifuged at 3,000 × g for 5 min. The pellet was resuspended in 3.8 μL of PBS (Phosphate-Buffered Saline) which was used as inocula.

Biofilm inocula were prepared by adjusting an overnight culture to OD_60__0__*n*__*m*_ = 1 ± 0.05 in TSB. The suspension was then diluted 1:100 and deposited in at least two flat-bottomed-wells in a 24-well cell culture plate (1 mL per well) before being placed in a humidity chamber at 37°C for 24 h without agitation. After the incubation period, the cell culture plate was overturned to eliminate excess media. Biofilms formed at the bottom of the wells were then steam-washed for 40 min as described previously (Tasse et al., 2018) and recovered by flushing and scraping the bottom of the well in 200 μL of PBS. This suspension was transferred to the second steam-washed well to recover biofilms, using the same recovery technique to obtain a final concentration of 5 × 10^6^ CFUs/3.8 μL of PBS. A small aliquot of the resulting suspension, 3.8 μL, was destined as inocula.

Bacterial CFUs in the injected inocula were confirmed by serial 10-fold dilutions on LB agar plates, containing erythromycin (10 μg/mL) for fluorescent strains, incubated overnight at 37°C. Before plating, biofilm inocula were first vortexed for 30 s, sonicated for 10 min (Thermo Fisher Scientific, 80 W, 37 kHz) and then vortexed again for a further 30 s. Average titrations of planktonic and biofilm inocula were 3.55 × 10^6^ ± 1.58 × 10^6^ CFUs/3.8 μL and 4.81 × 10^6^ ± 4.58 × 10^5^ CFUs/3.8 μL, respectively, with no significant difference between the values (results not shown).

Control inocula corresponded to a small volume of PBS, 3.8 μL.

### Micro-Injection of Biofilm and Planktonic Inocula Into Mice

Mice were anesthetized by intraperitoneal injection of a ketamine (100 mg/kg) and xylazine (5 mg/kg) mixture. Planktonic or biofilm inocula or PBS were micro-injected intradermally into the mouse ear pinna, as previously described (Mac-Daniel et al., 2016). Briefly, the ventral side of the mouse ear pinna was affixed under a stereomicroscope using clear tape. A 10 μL NanoFil syringe (World Precision Instruments) fitted to a 34-gauge beveled needle was then loaded with either 3.8 μL of planktonic or biofilm bacteria (containing 5 × 10^6^ CFUs) or PBS. Inocula were delivered into the ear tissue by inserting the needle beneath the epidermis on the dorsal side of the ear, and injecting the loaded suspension. A characteristic papule was observable at the injection site, evidence of an intradermal injection.

### Bacterial Burden in Ear Skin Tissue and Draining Lymph Nodes

The SH1000 WT strain was used for bacterial burden analysis. At 2 h pi, mice were euthanized, and the ear pinna tissue and auricular dLNs of infected and control mice were harvested using sterile scissors and weighed. Samples were kept on ice whenever possible throughout the duration of the experiment. Ear tissue and dLNs were first placed in 500 μL of sterile physiological water per ear or dLN, in individual tubes. Ear tissue were then washed in a bath of ethanol and then rinsed in two consecutive baths of distilled water before being dissected into small pieces and placed in a hemolysis tube containing 500 μL of sterile PBS. Draining lymph nodes were washed in two baths of distilled water and directly placed in a hemolysis tube containing 500 μL of sterile PBS. Ears and dLNs were homogenized using a PRO 200 handheld tissue homogenizer (PRO Scientific). Serial 10-fold dilutions were performed on tissue homogenates and plated on LB agar plates to enumerate viable *S. aureus* per milligram of tissue. Ear tissues and dLNs from mice inoculated with biofilm bacteria were vortexed and sonicated as described above before serial dilution and plating. Experiments were repeated from day 1 to day 7 pi and at 10 days pi.

### Evaluation of Ear Swelling

Ear swelling measurements were carried out on mice inoculated with SH1000 WT strain. Mice destined to be euthanized were first anesthetized by intraperitoneal injection of a ketamine and xylazine mixture. Ear thickness measurements were carried out in triplicate by the same experimenter using an engineer’s micrometer (Powerfix Profi Digital Caliper). Care was taken to measure only the outer two-thirds of the ear pinna and to not overly compress the swollen ear tissue between measurements. Due to the development of tissue necrosis at the later stages of infection, this parameter was only measured during the first 48 h pi.

### Inflammation Scoring

Upon mice euthanasia, photos were taken of the ear pinna tissue and then examined in a blinded fashion. Inflammation was scored on the basis of erythema (redness due to capillary swelling) ranging between 0 and 2. Ear tissue not presenting any signs of erythema were assigned a score of 0, while ears with any observable redness were scored between 1 and 2. The former corresponded to the presence of mild erythema over a small area of the ear tissue, while the latter referred to intense redness occupying a larger surface area on the skin.

### Multiplex Cytokine Assay

An aliquot from ear pinnae tissue and dLN homogenates generated during bacterial load experiments were centrifuged for 10 min at 10,000 × g at 4°C. Supernatants were stored with 1X Pierce protease inhibitor (Thermo Fisher Scientific) at −80°C until ready for analysis. Samples destined to a multiplex cytokine assay were first thawed on ice and centrifuged a second time for 10 min at 10,000 × g at 4°C. Right and left ear pinna tissue and dLNs from the same mouse were pooled before analysis. The cytokines tested in supernatants included murine IFN-γ, IL-12 p70, TNF-α, IL-17A, IL-6, IL-10, IL-1β, CXCL1, and CXCL9, and were analyzed using multiplex fluorescent bead arrays from Biorad on a BioPlex 200 Luminex system (BioRad), according to manufacturer’s directions. Concentrations were expressed per mg of total protein previously established by Bradford assay (Bio-Rad).

### Intravital Imaging Acquisition by Confocal Microscopy

#### Time-Lapse Video Acquisition

Two to five hours after inoculation of either planktonic or biofilm mCherry-SH1000 bacteria into the ear tissue of LysM-EGFP transgenic mice, mice were anesthetized to observe the recruitment of EGFP + fluorescent immune cells at the injection sites (intra vital imaging). Video time-lapse acquisition was carried out as previously described (Abdul Hamid et al., 2020). Briefly, the inner side of the ear pinna was flattened on a glass slide and imaged on the ZEISS Spinning Disk Cell Observer (SD) (Carl Zeiss Microscopy) confocal microscope. Acquisition was performed with 10X (dry), 20X (dry), and 40X (oil) objectives for periods of 20–30 min. Ear tissues of control mice were inoculated with PBS and imaged at the same time points.

#### Mosaic Acquisition

Infected ears were also imaged on the ZEISS LSM 800 (LSM 800) (Carl Zeiss Microscopy), 3–5 h after inoculation, as previously described (Abdul Hamid et al., 2020). Briefly, multiple fields of observation covering the entirety of the ear tissue surface were imaged with a 10X (dry) objective in order to reconstruct an image of the ear. EGFP fluorescence signal was detected in six Z-stacks while the bright-field signal was only detected on a central stack. Acquisition was repeated 24 and 48 h pi, with imaging sessions typically lasting 30–40 min. Ear tissues of control mice injected with PBS were also imaged with the same protocol.

### *In vivo* Confocal Image Analysis

#### SD Image and Video Analysis

Time-lapse videos at 20X and 40X were analyzed with Imaris software, as previously described (Abdul Hamid et al., 2020). Briefly, tracks were generated and attributed to each immune cell in the observation field using the “Spots” tool. Three different parameters of immune cell dynamics were then extracted: average speed, straightness and displacement. All cells in time-lapse videos where bacteria were visible were analyzed. For cell perimeter analysis, EGFP + immune cells destined to be measured were first selected from 40X time-lapse videos where bacteria were visible using the ZEN 3D image viewer. Only cells with clearly delimited borders present between the first and last stacks were chosen. ROIs were then drawn on Z-projected images prior to perimeter measurements using ImageJ software.

#### Mosaic Analysis

Images acquired on the ZEISS LSM 800 confocal were stitched together using ZEN software to reconstruct an entire image of the ear tissue at each time point. The images shown represent the Z-projected maximal intensity signal of a reconstituted image of the ear tissue for the EGFP channel. Images were then analyzed as previously described (Abdul Hamid et al., 2020). Briefly, a Region Of Interest (ROI) was drawn manually around the EGFP fluorescent zone of the 48-h image to obtain the sum of EGFP fluorescence intensities of each pixel in the ROI. The same ROI was applied to the other images of the same time point image and the ratio of the sum of intensities of EGFP fluorescence to the area of the ROI was then calculated for both time points.

### Lymph Node and Ear Cell Preparation for Flow Cytometry Analysis

The GFP-SH1000 fluorescent strain was used for flow cytometry analyses. At 2, 24, and 48 h pi, mice were euthanized, and the ear tissue and dLNs of infected and control mice were harvested using sterile scissors and weighed. Samples were kept on ice whenever possible throughout the duration of the experiment. Left and right ear pinna leaflets were first dissociated before being completely submerged in 5 mL of RPMI 1640 medium containing 1 mM HEPES, 400 U/mL collagenase IV and 50 μg/mL DNase I from bovine pancreas and placed at 37°C for 1 h. Left and right LNs were digested in the same type of medium but only for 15 min. After 15 min of incubation, ear pinna tissue was dissected into small pieces to facilitate digestion. Tissues were then ground on a 70-μm cell strainer to obtain single-cell suspensions before being centrifuged at 300 × g for 10 min at 4°C. Cell pellets were suspended in 500 μL of PBS containing 1% SVF and 1 mM Ethylenediaminetetraacetic acid (EDTA). Cells were then counted and labeled according to manufacturer’s directions, using the following markers: Viobility Fixable Dye, CD3ε, CD19, CD335, CD45, CD11b, Ly6G, and Ly6C. To define inflammatory cell populations recruited to these tissues, live single cells were gated to exclude any debris, dead cells or doublets. T lymphocytes, B lymphocytes, NK cells, and dead cells (DUMP channel) were excluded from live single cells, respectively, on the basis of the CD3ε, CD19, CD335, and Viobility 405/452 fixable dye specific markers. Live myeloid populations (CD45^+^CD11b^+^) were subdivided into PMNs (Ly6G^+^Ly6C^+^) and MO/MΦ (Ly6G^–^Ly6C^*hi*^). Samples were run on a BD LSR II (BD Biosciences), and data were analyzed with FlowLogic version 7.2.

### *Ex vivo* Intracellular Bacteria Analysis

The mCherry-SH1000 fluorescent strain was used for *ex vivo* intracellular bacteria analyses. At 2, 24 and 48 h pi, infected mice were euthanized, and the ear pinna tissue were harvested using sterile scissors and weighed. Samples were kept on ice whenever possible throughout the duration of the experiment. Left and right ear pinnae leaflets were first dissociated before being completely submerged in 700 μL of PBS containing Liberase^TM^ Research Grade (Roche), diluted 2:7, and placed at 37°C for 1 h with agitation. Fifteen mL of cold PBS were added to each tube and tissues were then ground on a 40-μm cell strainer to obtain single-cell suspensions that were then centrifuged at 1200 × g for 5 min at 4°C. Cell pellets were suspended in 1 mL of PBS containing 5% SVF. Cell suspensions resulting from 2 infected mice per bacterial form were pooled for 24- and 48-h time points. CD11b^+^ cells were isolated using CD11b microbeads mouse/human magnetic cell separation (Miltenyi Biotec) according to manufacturer’s directions.

To assess the quantity of intracellular bacteria, a portion of sorted skin cells were centrifuged at 300 × g for 10 min at 4°C, and resuspended in 1 mL of room temperature RPMI 1640 medium containing 100 μg/mL gentamycin. Cells were incubated for 1 h at 37°C, CO_2_ 5% before being centrifuged again at 300 × g for 10 min at 4°C. Cells were then lysed by resuspending pellets in 500 μL of cold ultrapure water, followed by vigorous shaking for 1 min. Serial 10-fold dilutions were performed on the resulting suspension and plated on LB agar plates to enumerate viable *S. aureus* per 10^6^ CD11b + cells.

To characterize the distribution of intracellular bacteria, another portion of sorted skin cells were adhered to glass slides by centrifugation (800 rpm for 5 min, Thermo Shandon Cytospin 3 Centrifuge) and were fixed with 4% PFA prior to MGG staining. Glass slides were then examined by bright field microscopy (Axio Imager 2, Zeiss) and intracellular bacteria were counted in at least 100 CD11b^+^ cells per condition, per time point.

### Neutrophil NADPH Oxidase Assay

The SH1000 WT strain was used for Neutrophil NADPH oxidase analysis. Single cell suspensions were obtained 48 h pi from mouse ear pinna tissue, as described in the flow cytometry analysis section. NADPH oxidase quantity was assessed, as described previously (Borbón et al., 2019). Briefly, cells were labeled using the following markers: CD45, CD11b, and Ly6G and then incubated for 15 min at 37∘C, 5% CO_2_ in PBS containing 10 μM of dihydrorhodamine 123 (DHR) (Sigma-Aldrich) with or without 100 ng/mL of phorbol myristate acetate (PMA) (Sigma-Aldrich). Samples were run on a BD LSR II (BD Biosciences), and data were analyzed with FlowLogic version 7.2.

### Statistical Analysis

Data generated were analyzed with Prism 5 software (GraphPad Software, Inc.) and a non-parametric Mann-Whitney one-tailed or two-tailed statistical test. *p* ≤ 0.05 was considered statistically significant (symbols: ^∗^ ≤ 0.05).

## Results

### Comparable Bacterial Load and Inflammatory Responses in the Mouse Ear Pinna After the Micro-Injection of Either the Planktonic or Biofilm Form of *Staphylococcus aureus*

**FIGURE 1:**
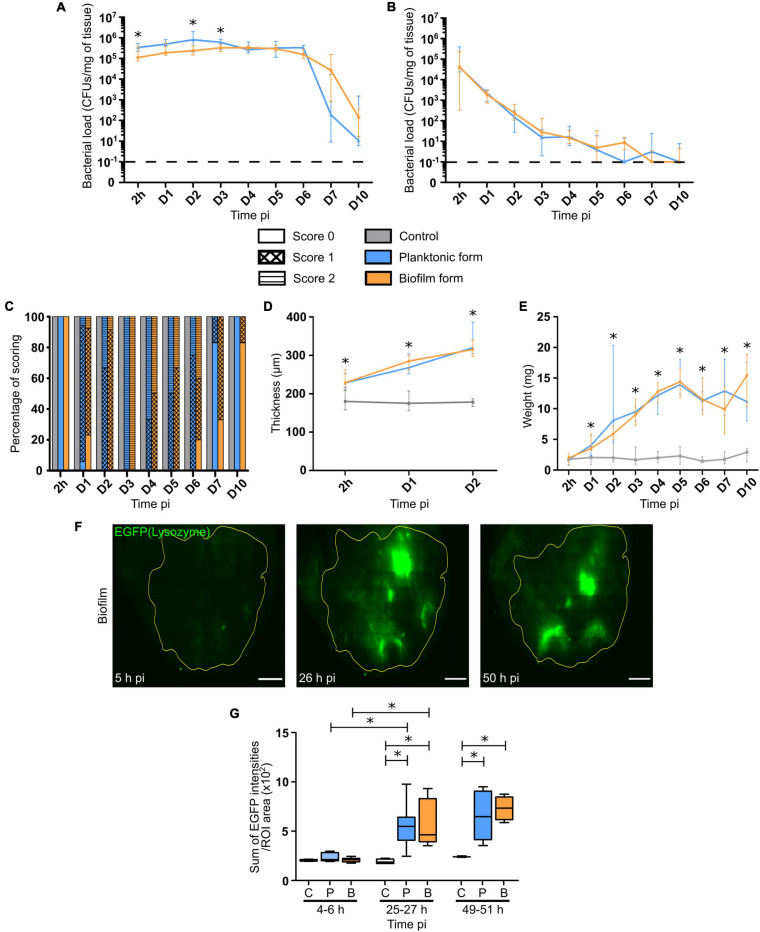
Bacterial load and inflammatory responses in *S. aureus* infected tissue. **(A,B)** Bacterial load quantification, expressed in CFUs/mg of tissue, from 2 h pi to day 7 pi, and at day 10 pi in the ear pinnae **(A)** and dLNs **(B)** of WT C57BL/6J mice following micro-injection of PBS (C) or WT SH1000 planktonic (P) or biofilm (B) bacteria. Dotted line represents the limit of detection (median ± IQR, number of ear pinnae: N_*C*_ = 8–10 ear pinnae, N_*P*_ = 10–12 ear pinnae, N_*BF*_ = 11–12 ear pinnae, from 3 different experiments, Mann-Whitney two-tailed test, ^∗^*p* < 0.05). **(C)** Evaluation of ear pinnae inflammation from 2 h pi to day 7 pi, and at day 10 pi for the 3 groups of mice, by scoring based on erythema. Results expressed as percentage of each score (Number of mice: N_*C*_ = 6–10 mice, N_*P*_ = 4–17 mice, N_*BF*_ = 5–13 mice, from 3 different experiments). **(D)** Ear pinna thickness measurements from 2 h pi to day 2 pi for the 3 groups of mice (median ± IQR, number of ear pinnae: N_*C*_ = 4–7 ear pinnae, N_*P*_ = 6–8 ear pinnae, N_*BF*_ = 6–7 ear pinnae, from 3 different experiments, Mann-Whitney two-tailed test, ^∗^*p* < 0.05). **(E)** dLN weight measurements from 2 h pi to day 7 pi, and at day 10 pi for the 3 groups of mice (median ± IQR, number of dLNs: N_*C*_ = 8–10 dLNs, N_*P*_ = 10–12 dLNs, N_*BF*_ = 11–12 dLNs, from 3 different experiments, Mann-Whitney two-tailed test, ^∗^*p* < 0.05). **(F)** Reconstituted confocal images of LysM-EGFP transgenic mouse ear pinna tissue following micro-injection of mCherry-SH1000 biofilm bacteria. Images correspond to the maximal projection intensities of the EGFP signal, and the yellow line indicates the ROI where the “Sum of EGFP fluorescence intensities” was measured. Scale bar: 2 mm. One representative image is shown from at least 3 independent experiments. **(G)** Ratio of the sum of EGFP fluorescence intensities to ROI areas (median ± IQR, number of ear pinnae tissues: N_*C*_ = 4, N_*P*_ = 5–8, N_*BF*_ = 4–9, from at least 3 different experiments, Mann-Whitney two-tailed test, ^∗^*p* < 0.05).

Calibrated *S. aureus* inocula were prepared and microinjected into the mouse ear pinna tissue. Bacterial load was first measured in the cutaneous ear tissue and the auricular draining lymph node (auricular dLN) following inoculation of WT C57BL/6J mice with 5 × 10^6^ CFUs of non-fluorescent planktonic or biofilm *S. aureus*. CFUs were enumerated, starting from 2 h post-infection (h pi) until day 7 pi, and then after 10 days. Bacterial load quantification showed that viable planktonic and biofilm bacteria were detected in both cutaneous ear tissue and dLNs at 2 h pi (Figures 1A,B), and were continuously present throughout the infection period. In the ear pinna tissue, bacterial loads were mostly similar and stable throughout the first 6 days of infection for both forms of bacteria, with the first signs of bacterial clearance appearing at day 7. Although this sharp decrease seemed more pronounced in mice inoculated with planktonic bacteria, no significant difference was observed between the two forms. At day 10 pi, a lower bacterial load was detected in both groups of infected mice, as compared to day 7 pi, but was once again comparable between the two forms. In the dLNs, bacteria were present throughout all the infection period, with similar infection kinetics. This was in line with the maintenance of bacteria in the cutaneous tissue previously observed. Indeed, CFUs were detected even at day 10 pi in the dLN in 3/10 and 3/12 samples for planktonic and biofilm bacteria, respectively. At this time point, the bacterial load was low in most of the samples and often below the detection threshold.

Certain macroscopic parameters of inflammation related to the presence of bacteria in the target tissues (ear pinnae, dLNs) were then analyzed, including redness and swelling (thickness) of ear pinna tissue and dLN weight. In control mice, no redness was observed throughout the experimental period (Figure 1C). The first signs of redness (Supplementary Figure 1) appeared 24 h pi within the infected group of mice (Figure 1C), where most samples had a score of 1. Redness then peaked at day 3 pi within both infected groups, and then slowly decreased to give way to tissue necrosis from day 2 pi, with a peak at day 10 pi. Interestingly, the presence of redness in the cutaneous ear tissue lasted longer in the biofilm group of mice than in the planktonic group. The ear tissue swelling was further analyzed in the first 2 days of infection. Ear thickness was significantly increased as early as 2 h pi, as compared to control mice. Values continued to increase until the second day of infection with no observable difference between the two infected groups of mice (Figure 1D). Lastly, the weight of dLNs significantly and constantly increased after infection, as compared to control mice, starting from day 1 pi until the end of the infection period. Again, no observable difference was observed between the two infected groups of mice (Figure 1E).

To follow the evolution of early inflammatory responses in the skin, a semi-quantitative measure of EGFP positive (EGFP +) phagocyte recruitment in the ear pinna tissue was realized as previously described (Abdul Hamid et al., 2020; Figure 1F and Supplementary Figure 2). The ratio of the sum of EGFP fluorescence intensities to ROI area (Figure 1G), measured from 2 to 48 h pi, showed that both bacterial forms did not induce a significant increase in EGFP + cell recruitment at early time points (4–6 h pi), as compared to control mice. However, after 25 and 49 h pi, a significant increase of the ratio was observed in both groups of challenged mice, as compared to control mice. In infected mice, a significant increase of cell recruitment was observed between 4–6 and 25–27 h pi, but not between 25–27 and 49–51 h pi, meaning that the peak of cell recruitment was on day 1.

Taken together, these data show that global inflammatory responses are comparable in the target tissues after micro-injection of both bacterial forms.

### Comparable Early Cytokine and Chemokine Profiles in the Mouse Ear Pinna and the Auricular Draining Lymph Node After the Micro-Injection of Either the Planktonic or Biofilm Form of *Staphylococcus aureus*

In a second set of experiments, cytokine and chemokine profiles were analyzed in ear tissue and dLN homogenates during the first few days of infection (24–72 h pi).

**FIGURE 2:**
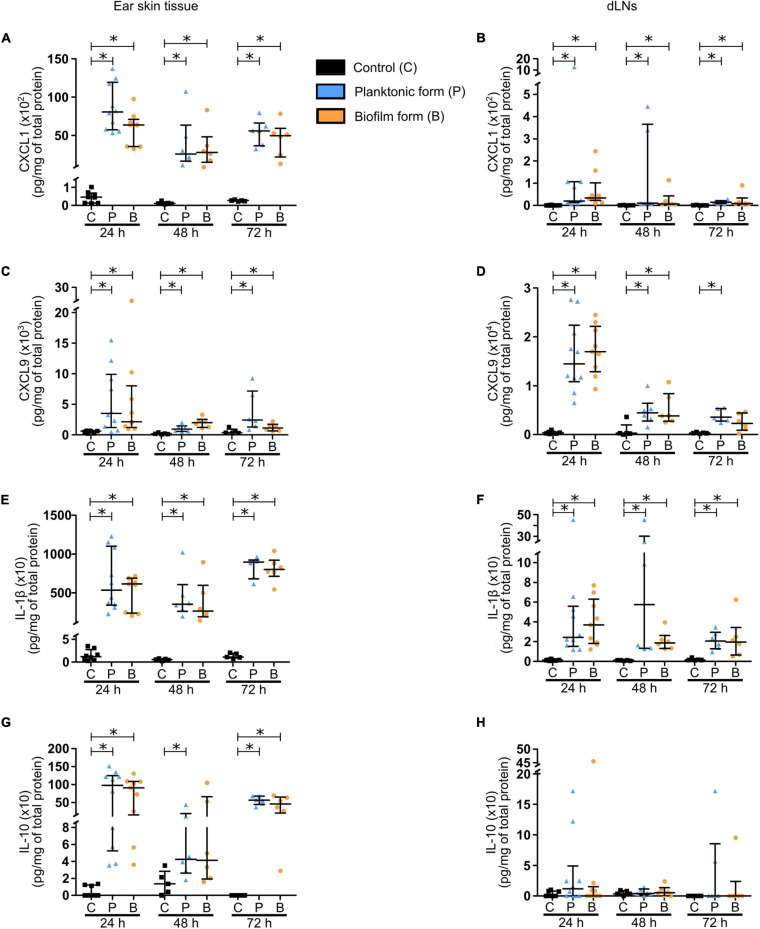
Chemokine and cytokine production in *S. aureus* infected tissue. **(A–H)** Chemokine **(A–D)** and cytokine **(E–H)** levels, expressed in pg/mg of total protein, analyzed by Bioplex in the supernatants of ear pinna tissues **(A,C,E,G)** and dLNs **(B,D,F,H)** homogenates at 24, 48, and 72 h pi (median ± IQR, number of mice: N_*C*_ = 5–8, N_*P*_ = 6–10, N_*BF*_ = 6–9, from at least 3 different experiments, Mann-Whitney two-tailed test, ^∗^*p* < 0.05).

The chemokines analyzed were CXCL1/KC (Figures 2A,B) and CXCL9/MIG (Figures 2C,D), both responsible for the recruitment of phagocytes, namely PMNs and MOs/MΦs. Significantly higher levels of these chemokines were detected from 24 to 72 h pi in both target tissues, with higher concentrations measured in the skin than in the dLNs of infected mice. Globally, no difference was observed between the two bacterial forms, except after 72 h in the dLN where CXCL9 were comparable to those in controls.

The chemokine response was associated with a pro-inflammatory cytokine production by recruited innate immune cells. Pro-inflammatory cytokines IL-1β (Figures 2E,F) and IL-6 (Supplementary Figures 3A,B) were significantly increased in both target tissues, again with higher concentrations measured in the skin than in the dLNs of infected mice, and at all-time points analyzed. A larger quantity of TNF-α was also detected in the ear pinnae of infected mice, as compared to controls at 24 and 72 h pi (Supplementary Figure 3C). However, this increase was only observed at 24 h pi in the dLNs (Supplementary Figure 3D). Interestingly, IFNγ concentrations were comparable in control and infected mice in cutaneous ear tissue at 24 and 72 h pi (Supplementary Figure 3E), but were significantly increased at both time points in dLNs of infected mice, as compared to those of control mice (Supplementary Figure 3F). The last pro-inflammatory cytokine analyzed was IL-17 (Supplementary Figures 3G,H), largely produced by Th17 cells and capable of upregulating the expression of certain proinflammatory mediators such as IL-1β and IL-6 in MΦs. As seen previously for IL-6, significantly higher levels of IL-17 were detected in both target tissues of infected mice, as compared to controls at 24 and 72 h pi.

Finally, concentrations of the anti-inflammatory cytokine IL-10 (Figures 2G,H) were significantly increased in the ear pinnae tissue of infected mice, as compared to control mice at 24 and 72 h pi, while only being significantly increased after inoculation of planktonic bacteria at 48 h pi.

Altogether, these results reveal early pro-inflammatory and anti-inflammatory responses in both target tissues at the molecular level, with IL-1β, IL-10, and CXCL1 being majorly produced in the cutaneous ear tissue, and CXCL9 largely detected in the dLN. These responses were globally comparable for both bacterial forms.

### Differential Early Dynamics of Recruited Phagocytes in the Mouse Ear Pinna After the Microinjection of Either the Planktonic or Biofilm Form of *Staphylococcus aureus*

The very early dynamics of inflammatory responses observed in the skin were further analyzed by using an intravital confocal imaging approach, as previously described (Abdul Hamid et al., 2020). The first goal was to reproduce our previous results described for the *S. aureus* LYO-S2 clinical strain with the SH1000 laboratory strain in terms of cell recruitment and behavior of recruited cells after biofilm infection. Briefly, 5 × 10^6^ CFUs of planktonic or biofilm mCherry-SH1000 were intradermally inoculated in the ear pinna tissue of LysM-EGFP transgenic mice. Time-lapse videos of cutaneous injection sites were then acquired during the first few hours following infection (from 2 to 5 h pi).

**FIGURE 3:**
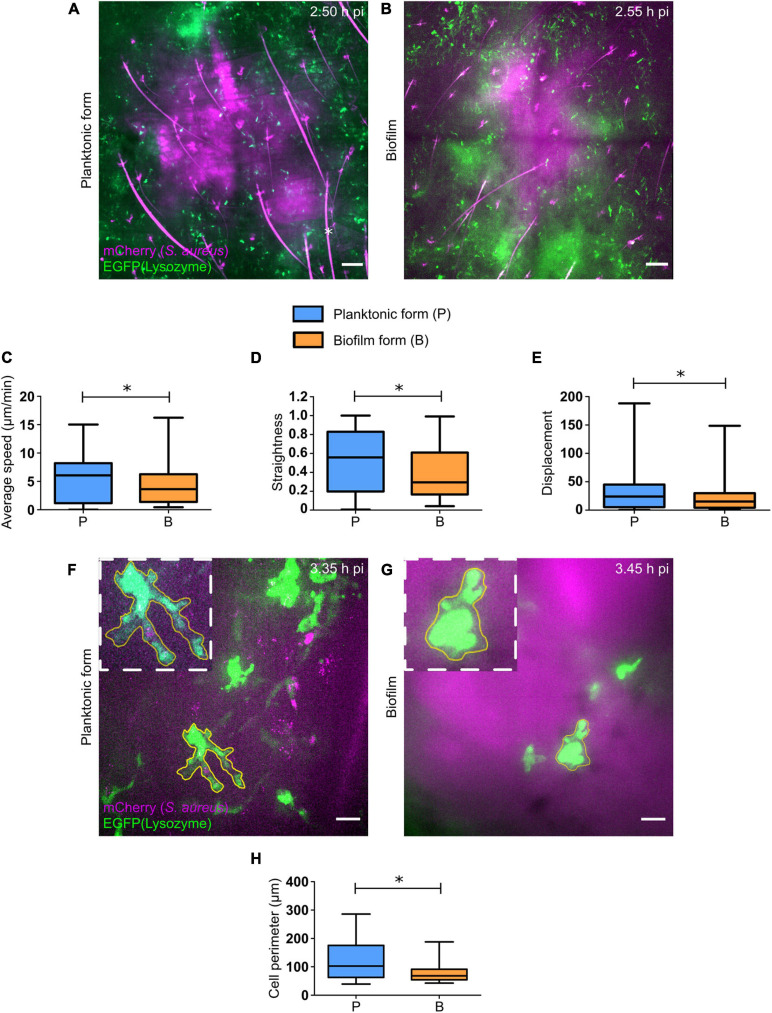
Early dynamics of recruited EGFP + cells in the skin of *S. aureus* infected tissue. **(A,B)** Live confocal imaging, using X10 objectives, in the ear pinna tissue of LysM-EGFP transgenic mice following micro-injection of mCherry-SH1000 planktonic **(A)** or biofilm **(B)** bacteria. Average projections of green (EGFP + innate immune cells) and magenta (mCherry-SH1000) fluorescence, acquired at 2.50 h pi **(A)** and 2.55 h pi **(B)**, show immune cells recruited toward the injection site. Asterisk: autofluorescent hair (also in magenta). Scale bar: 100 μm. One representative experiment is shown for each group of mice from at least 9 independent experiments. **(C–E)** Quantification of EGFP + innate immune cell average speed **(C)**, straightness **(D)**, and displacement **(E)**, from X20 time-lapse acquisitions of infected mice (median ± minimum and maximum values, number of cells: N_*P*_ = 209, N_*BF*_ = 126, from at least 3 different experiments, Mann-Whitney two-tailed test, ^∗^*p* < 0.05). **(F,G)** Live confocal imaging, using X40 objectives, in the ear pinna tissue of LysM-EGFP transgenic mice following micro-injection of mCherry-SH1000 planktonic **(F)** or biofilm **(G)** bacteria. Maximum projections of green (EGFP + innate immune cells) and magenta (mCherry bacteria) fluorescence, acquired at 3.35 h pi **(F)** and 3.45 h pi **(G)**, show immune cells interacting with bacteria. The yellow line indicates the ROI where cell perimeter was measured. Scale bar: 15 μm. One representative experiment is shown for each group of mice from at least 9 independent experiments. **(H)** Measure of EGFP + innate immune cell perimeter, from maximum projection time-lapse acquisitions of infected mice (median ± minimum and maximum values, number of cells: N_*P*_ = 19, N_*BF*_ = 25, from 4 different experiments, Mann-Whitney one-tailed test, ^∗^*p* < 0.05).

Using this laboratory strain, we observed a considerable influx of EGFP + phagocytes at injection sites, as early as 2 h pi for both bacterial forms. Cells recruited in response to the planktonic form were capable of invading the injection sites and creating bacteria cell interaction zones (Figure 3A, Supplementary Figures 4A,B, and Supplementary Movie 1). In contrast, cells that were recruited toward biofilm injection sites behaved differently, as previously described (Abdul Hamid et al., 2020). Indeed, EGFP + innate immune cells were arrested at the periphery of biofilm injection sites, with only few cells capable of penetrating the biofilm (Figure 3B, Supplementary Figures 4C,D, and Supplementary Movie 2).

The motility parameters (average speed, trajectory straightness, displacement) of recruited EGFP + phagocytes both in contact or not with bacteria at the injection site were also studied using Imaris software, as previously described (Abdul Hamid et al., 2020). SH1000 biofilms impacted cell motility similarly to previous observations, with a significant decrease in average speed and straightness of cells recruited toward biofilm bacteria, as compared to the planktonic form (Figures 3C,D). This means that cells were more heavily arrested and had a non-linear trajectory in biofilm-inoculated sites, as compared to planktonic-inoculated sites. The displacement of cell trajectories, representing the straight-line distance of the cell from the first timepoint to the last, was also analyzed in both infected conditions (Figure 3E). A significant decrease of this parameter was detected following biofilm inoculation, as compared to planktonic condition. This result was coherent with the decrease in straightness of cell trajectories observed with biofilm inocula.

Interestingly, observations made during intravital confocal microscopy experiments revealed that cells in contact with biofilms behaved differently from those in contact with planktonic bacteria, with a diminished capacity to emit pseudopods and retaining a round morphology (Figures 3F,G and Supplementary Figures 4E–H). Quantitative data were obtained by measuring the perimeter of EGFP + phagocytes in contact with either bacterial form with ImageJ software. Using this methodology, a significant decrease of cell perimeter in the presence of the biofilm inoculum was observed, as compared to planktonic inocula (Figure 3H). This result obtained *in vivo* was coherent with observations made *in vitro*, where PMNs in contact with *Pseudomonas aeruginosa* biofilms conserved their round shape (Jesaitis et al., 2003).

### Differential Kinetics of Polymorphonuclear Neutrophil Recruitment After the Micro-Injection of Either the Planktonic or Biofilm Form of *Staphylococcus aureus*

**FIGURE 4:**
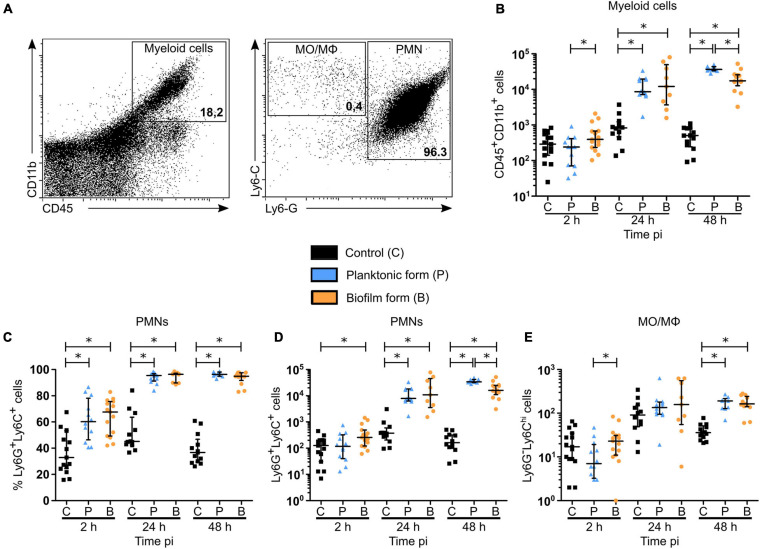
Myeloid cell recruitment in the skin of *S. aureus* infected tissue. **(A)** Flow cytometric analysis showing the gating strategy of myeloid cells (CD45^+^CD11b^+^, left plot—upper right region), PMNs (Ly6G^+^Ly6C^+^, right plot—right most region), MOs/MΦs (Ly6G^–^ Ly6C^*hi*^, right plot—upper left). Representative dot plots and percentages of cells gated are shown from planktonic infected WT C57BL/6J mice at 48 h pi. **(B)** Total number of myeloid cells among live cells in ear pinna tissue of control and planktonic or biofilm infected mice from 2 h pi to day 2 pi (median ± IQR, number of mice: N_*C*_ = 12–15, N_*P*_ = 9–12, N_*BF*_ = 9–15, from at least 3 different experiments, Mann-Whitney two-tailed test, ^∗^*p* < 0.05). **(C–E)** Percentages **(C)** and total numbers **(D,E)** of PMNs **(C,D)** and MOs/MΦs **(E)** among myeloid cells in ear pinnae of control and planktonic or biofilm infected mice, from 2 h pi to day 2 pi (median ± IQR, number of mice: N_*C*_ = 12–15, N_*P*_ = 9–12, N_*BF*_ = 9–15, from at least 3 different experiments, Mann-Whitney two-tailed test, ^∗^*p* < 0.05).

To further understand the early inflammatory responses to the biofilm inocula, imaging data were complemented by flow cytometry analysis of the phenotype of recruited inflammatory cells in the cutaneous ear tissue and the dLNs. Here, ear pinna tissue of WT C57BL/6J mice were inoculated with either PBS or 5 × 10^6^ CFUs of GFP fluorescent *S. aureus* planktonic or biofilm bacteria. After 2, 24, and 48 h, organs were harvested, digested to obtain a single cell suspension, labeled for specific membrane markers of innate inflammatory cells, and then analyzed by flow cytometry. Myeloid populations (CD45^+^CD11b^+^) were identified among live single cells and further subdivided into PMNs (Ly6G^+^Ly6C^+^) and MO/MΦ (Ly6G^–^Ly6C^*hi*^) (Figure 4A and Supplementary Figure 5).

Total cell counts prior to labeling showed a significant recruitment of cells to the cutaneous ear tissue as soon as 2 h pi in infected mice, as compared to control mice, which continued until 48 h pi (Supplementary Figure 6A). However, absolute values of myeloid cells in the ear pinna tissue of infected mice were only significantly increased after 24 and 48 h, as compared to control mice (Figure 4B). These data correlate with the previous measures of overall tissue inflammation in LysM-EGFP transgenic mice (Figure 1G). Interestingly, a decrease in the number of myeloid cells recruited in the ear tissue was observed 48 h following biofilm inoculation, as compared to the planktonic condition.

When looking further at the phenotype of recruited myeloid cells, a significantly higher percentage of PMNs was present in infected cutaneous ear tissue compared to controls, from 2 to 48 h pi (Figure 4C). However, PMN cell numbers revealed that this increase was significant only after 24 h in the planktonic group of mice, and from 2 h pi in biofilm infected mice (Figure 4D). Indeed, biofilms recruited more PMNs than controls at 2 h pi, with only a trend to increase when compared to mice infected with planktonic bacteria (*p* = 0.0673). Similar to their parent population (CD45^+^CD11b^+^ cells), biofilms recruited a significantly lower number of PMNs at 48 h pi, as compared to planktonic inoculated sites.

Altogether, Figures 4C,D illustrate that the majority of myeloid cells, and thus EGFP + phagocytes recruited to the cutaneous ear tissue after biofilm injection were PMNs. Their recruitment kinetics were different at 2 and 48 h pi, when compared to planktonic bacteria.

For MO/MΦ, a significant recruitment was detected in ear pinna tissue for both bacterial forms only after 48 h, as compared to control mice, but in a much lower proportion compared to PMNs (Figure 4E).

**FIGURE 5:**
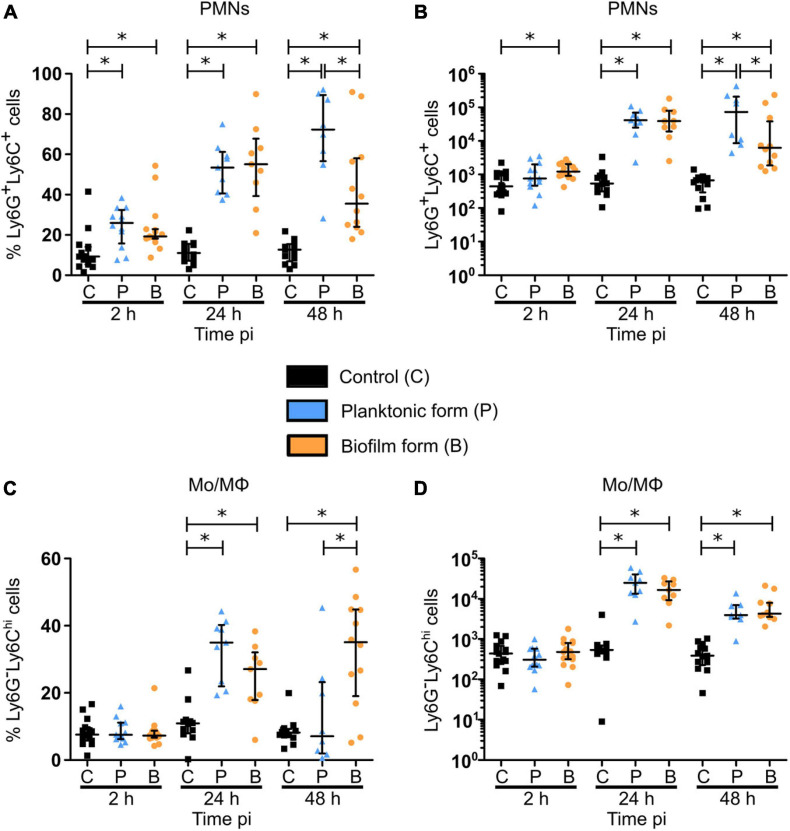
Myeloid cell recruitment in the auricular draining lymph nodes of *S. aureus* infected mice. **(A–D)** Percentages **(A,C)** and total numbers **(B,D)** of PMNs **(A,B)** and MOs/MΦs **(C,D)** among myeloid cells in dLNs of control and planktonic or biofilm infected mice, from 2 h pi to day 2 pi (median ± IQR, number of mice: N_*C*_ = 12–15, N_*P*_ = 8–12, N_*BF*_ = 9–15, from at least 3 different experiments, Mann-Whitney two-tailed test, ^∗^*p* < 0.05).

In dLNs, total cell counts were significantly increased only after 48 h with planktonic bacteria, as compared to controls, while only a trend to increase was detected in the biofilm group of mice (*p* = 0.069) (Supplementary Figure 6B). As shown previously in the cutaneous ear tissue, a significant increase in the percentage of PMNs was observed at 2 and 24 h pi in biofilm infected mice, as compared to control mice (Figure 5A). Additionally, a decrease in the percentage of these cells among their parent population was observed after 48 h, as compared to the planktonic condition. Thus, recruitment kinetics of PMNs in the dLN were similar to those in the cutaneous ear tissue (Figure 5B). Interestingly, bacteria inoculation induced a significant increase in both percentage and MO/MΦ cell numbers in the dLN at 24 and 48 h pi, a result which was not observed in the cutaneous ear tissue (Figures 4E, 5B,C). In contrast to PMNs, the percentage of MO/MΦ in the dLN was higher after 48 h following biofilm inoculation, as compared to the planktonic condition (Figures 5A,C).

Taken globally, these results show that PMNs comprise the majority of cells recruited during the early stages of anti-biofilm inflammatory responses. However, their recruitment seemed to be specifically impacted by biofilms.

### *Staphylococcus aureus* Biofilm Bacteria Inoculated Into the Mouse Ear Pinna Mostly Interact With Polymorphonuclear Neutrophils

**FIGURE 6:**
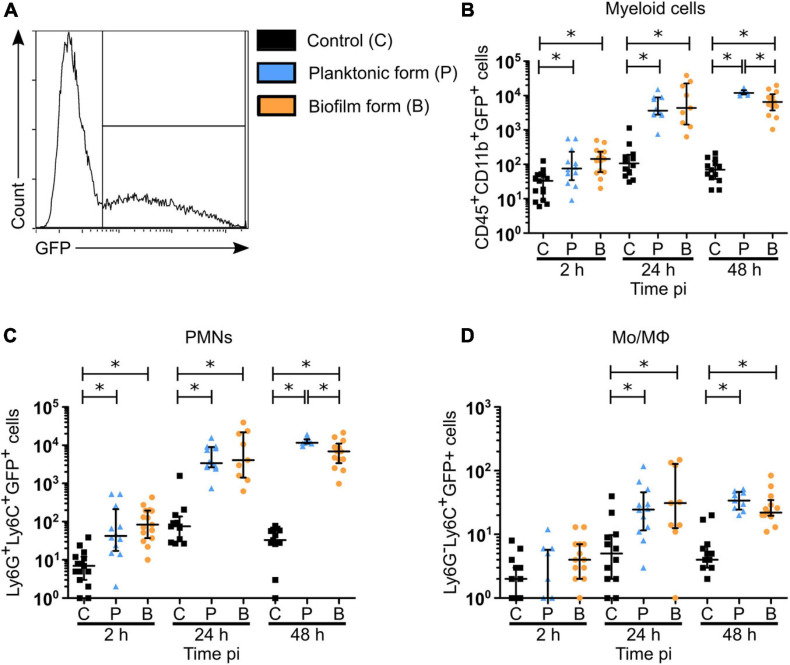
Biofilm bacteria associated myeloid cells in the skin of *S. aureus* infected mice. **(A)** Flow cytometric analysis showing the gating strategy to analyze GFP-SH1000 associated cells among myeloid cells (CD45^+^CD11b^+^GFP^+^), PMNs (Ly6G^+^Ly6C^+^GFP^+^), and MOs/MΦs (Ly6G^–^ Ly6C^*hi*^GFP^+^). Representative histogram is shown from planktonic infected WT C57BL/6J mice at 48 h pi. **(B–D)** Total number of bacteria associated myeloid cells **(B)**, PMNs **(C)**, and MOs/MΦs **(D)** in ear pinna tissue of control and planktonic or biofilm infected mice, from 2 h pi to day 2 pi (median ± IQR, number of mice: N_*C*_ = 12–15, N_*P*_ = 9–12, N_*BF*_ = 9–15, from at least 3 different experiments, Mann-Whitney two-tailed test, ^∗^*p* < 0.05).

Imaging data presented in Figure 3 illustrate qualitative differences in the interaction between EGFP + innate immune cells and either planktonic or biofilm bacteria at cutaneous injection sites. Thus, in complementary experiments, bacterial association with recruited inflammatory cells, and more specifically PMNs, was quantified in the cutaneous ear tissue by flow cytometry. To do this, ear pinnae of WT C57BL/6J mice were inoculated with either PBS or 5 × 10^6^ CFUs of planktonic or biofilm GFP-SH1000, and the proportion of recruited inflammatory cells associated to bacteria was quantified at 2, 24, and 48 h pi (Figure 6A). To verify the specificity of bacterial association, we measured the median fluorescence intensity (MFI) of the GFP signal in PMN and MO/MΦ subpopulations and compared them to the MFIs of controls (Supplementary Figures 6C,D). Supplementary Figures 6E,F show that although GFP autofluorescent cells were detected in ear tissues of control mice, a significant GFP specific signal was associated to recruited phagocytes in the ear tissues of infected mice (Supplementary Figures 6C–F).

Bacterial association was observed in myeloid cell populations (CD45^+^CD11b^+^) as early as 2 h pi (Figure 6B), which corroborates observations made by intravital imaging (Figure 3). The proportion of bacteria associated cells increased significantly for both bacterial forms at 24 and 48 h pi, as compared to PBS inoculated control mice. Interestingly, a decrease in biofilm association was observed at 48 h pi, as compared to planktonic bacteria. Recruited PMNs followed the same kinetics of bacterial association as myeloid cells (Figure 6C and Supplementary Figure 6E), with the same significant decrease observed after 48 h for the biofilm inoculum. These data were coherent with the decrease of myeloid cells and PMN numbers observed at this time point (Figures 4B,D). Bacteria associated to MOs/MΦs were detected in a significantly increased proportion at 24 and 48 h pi (Figure 6D and Supplementary Figure 6F), but in a much smaller proportion, as compared to PMNs. Furthermore, the percentage of PMNs associated to bacteria was continuously higher than those observed in MO/MΦ at all three time points analyzed.

Taken together, these data suggest that PMNs represent key cells in the interactions with biofilm bacteria at the cutaneous injection site.

### Differential Functional Properties of Recruited Polymorphonuclear Neutrophil in the Mouse Ear Pinna After the Micro-Injection of Either the Planktonic or Biofilm Form of *Staphylococcus aureus*

Figures 4–6 illustrated that PMNs consist of the majority of inflammatory cells recruited to both the cutaneous ear tissue and the dLN, and are greatly associated to biofilms at the cutaneous injection site. In complementary experiments, their functional properties were studied in the ear pinna tissue of WT C57BL/6J mice, after inoculation of either PBS or *S. aureus* planktonic or biofilm bacteria.

**FIGURE 7:**
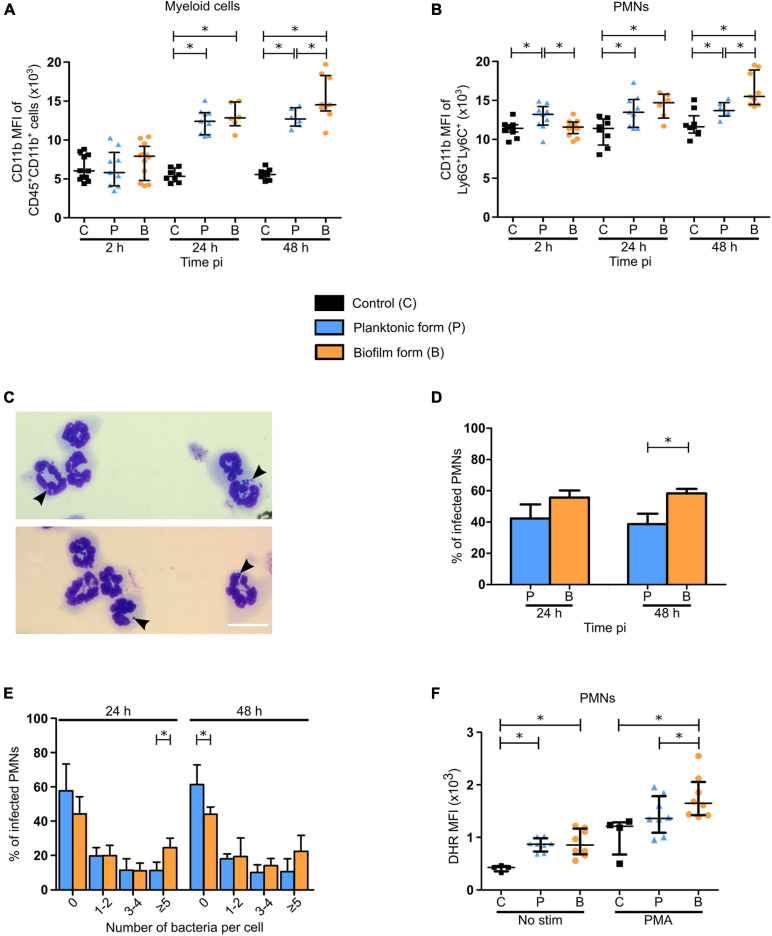
Functional properties of neutrophils recruited in the ear pinna tissue of *S. aureus* infected mice. **(A,B)** APC-Cy7 conjugated CD11b antibody MFI of myeloid cells (CD45^+^CD11b^+^) **(A)** and PMNs (Ly6G^+^Ly6C^+^) **(B)** in ear pinna tissue of control and planktonic or biofilm infected mice, from 2 h pi to day 2 pi (median ± IQR, number of mice: N_*C*_ = 8–11, N_*P*_ = 6–9, N_*BF*_ = 6–12, from at least 3 different experiments, Mann-Whitney two-tailed test, ^∗^*p* < 0.05). **(C)** Transmitted light images of CD11b^+^ cells, following isolation from ear pinna tissue onto glass slides and MGG staining, show neutrophils harboring intracellular bacteria. Representative images are shown from planktonic infected WT C57BL/6J mice at 24 h pi. **(D)** Percentage of PMNs containing intracellular bacteria assessed at 24 and 48 h pi (mean ± SEM, number of cells: N_*P*_ = 223–238, N_*BF*_ = 232–338, from at least 3 different experiments, Mann-Whitney one-tailed test, ^∗^*p* < 0.05). **(E)** Percentage of PMNs containing 0, 1–2, 3–4, or 5 or more intracellular bacteria assessed at 24 and 48 h pi (mean ± SEM, number of cells: N_*P*_ = 223–238, N_*BF*_ = 232–338, from at least 3 different experiments, Mann-Whitney one-tailed test, ^∗^*p* < 0.05). **(F)** DHR MFI of PMNs, in the ear pinna tissue of control and planktonic or biofilm infected mice at day 2 pi, stimulated or not with PMA *ex vivo* in the presence of DHR, to assess NADPH oxidase activity (median ± IQR, number of mice: N_*C*_ = 4, N_*P/BF*_ = 8, from at least 3 different experiments, Mann-Whitney one-tailed test, ^∗^*p* < 0.05).

In the first set of experiments, activation of myeloid cells and PMNs was assessed at 2, 24, and 48 h pi, based on their expression of the activation marker integrin receptor CD11b (Figures 7A,B). For both populations studied, an increased expression of CD11b was observed at 24 and 48 h pi, as compared to control mice, with a significant increase following biofilm inoculation at 48 h pi, as compared to the planktonic group. Interestingly, an initial significant increase in CD11b expression was shown 2 h pi in PMN populations only after planktonic bacteria inoculation.

In a second set of experiments, phagocytic activity of PMNs and MO/MΦ was analyzed 24 and 48 h pi. Intracellular bacteria were counted inside CD11b^+^ cells purified from cutaneous infected tissues and subsequently stained using the May Grünwald-Giemsa (MGG) staining (Figure 7C and Supplementary Figures 7A,B). This analysis revealed that the proportion of PMNs harboring intracellular bacteria was significantly increased at 48 h pi when biofilms were inoculated, as compared to planktonic bacteria (Figure 7D). Further analyses showed that the proportion of PMNs harboring 5 or more intracellular bacteria was significantly increased in biofilm infected mice compared to the planktonic condition after 24 h. This seemed to continue at 48 h pi where a trend to increase was observed in the same group of cells. Conversely, the proportion of PMNs not harboring any intracellular bacteria was significantly increased 48 h pi after planktonic bacteria inoculation (Figure 7E), illustrating differential phagocytosis capacities of PMNs in response to the planktonic or biofilm inocula.

For MO/MΦ, the proportion of intracellular bacteria was comparable for both bacterial forms at both 24 and 48 h pi (Supplementary Figure 7C). However, as seen previously for PMNs, the proportion of these cells harboring 5 or more intracellular bacteria was also significantly increased after 24 h for biofilm inocula (Supplementary Figure 7D).

The viability of intracellular bacteria counted on MGG stained slides was also verified (Supplementary Figure 7E). To do this, CD11b + cells were purified from ear skin tissue of infected mice and treated with gentamycin. Cells were then lysed, and bacteria were enumerated following plating on agar plates. Results indicate the presence of viable intracellular planktonic and biofilm bacteria inside these cells (mainly PMNs and MOs/MΦs) at 24 and 48 h pi, in similar proportions.

Figures 4C,D illustrate that PMN recruitment was significantly decreased in response to biofilms after 48 h, as compared to planktonic inocula. However, the increased expression of the CD11b surface marker and of phagocytosis capacity of PMNs observed at this time-point supposed that they were more activated in this context. To test this hypothesis, flow cytometry analysis was performed to assess DHR oxidation by Reactive Oxygen Species (ROS), reflective of NADPH oxidase activity generated in recruited PMNs in response to PMA (Figure 7F). When not in the presence of this soluble agent, DHR MFI was comparable between the two bacterial forms, despite being significantly increased compared to controls. PMA-stimulated PMNs from infected ear pinnae also presented a higher DHR MFI than controls, with an increased production of ROS after biofilm inoculation, as compared to planktonic bacteria.

Taken together, these results illustrated differential functional properties in recruited PMNs at 48 h pi following biofilm inoculation.

## Discussion

PMNs and MΦ have been largely described as key effector cells in the inflammatory responses during *S. aureus* infections (de Vor et al., 2020; Pidwill et al., 2021). Certain *in vivo* models have also shown that biofilms induce anti-inflammatory responses as early as day 3 or day 5 pi (Heim et al., 2015, 2018). As the infection outcome is conditioned in the first few days following host-bacteria contact, we sought out to highlight the very early impacts of *S. aureus* biofilms on the main cellular components of innate immune responses, meaning phagocytes. To do this, we compared inflammatory responses elicited by either planktonic or biofilm bacteria at different levels of the organism, in a mouse ear pinna model of infection. In this report, we demonstrated globally similar responses at the tissue and cytokine level, but found different cellular responses, including cell behavior, recruitment, and certain functional properties.

At the tissue level, we first observed a globally similar evolution of bacterial load between the two forms of *S. aureus*, contradicting a previous study that compared the fate of non-biofilm and biofilm inocula in mice. Thurlow and collaborators showed a better bacterial clearance of non-biofilm bacteria in skin abscess as early as day 3 compared to catheter grown biofilms implanted sub-cutaneously (Thurlow et al., 2011). They assigned this to the presence of the catheter, which could impede access of leukocytes toward the site of infection. In contrast, in the ear pinna model used in this work, biofilm inocula were accessible to host innate immune cells as no abiotic support was used. One possible reason for the comparable bacterial load observed in the ear pinna tissue could be the *in vivo* transition of planktonic bacteria to the biofilm lifestyle, representing a possible limit of the model. Several days are nevertheless probably necessary for this transition, even if the precise timing of this event to occur *in vivo* is currently unknown. In the clinical setting, *S. aureus* is prevalent in early prosthetic infections, which become manifest within 1 month following surgery (Arciola et al., 2018). To identify a possible planktonic to biofilm transition in the ear pinna, biofilm matrix components could be labeled *in vivo* by using specific fluorescent probes several days pi (Sauvat et al., 2020). Genes upregulated during biofilm formation could also be identified by transcriptomic analysis from planktonic-infected ear tissues, as previously carried out *in vitro* by Resch et al. (2005).

An observable inflammatory response was characterized by the apparition of redness and tissue swelling of the ear pinna tissue of infected mice, which was coherent with the presence of a high dose of bacteria in the first few days of infection. These results were further corroborated by the semi-quantitative measure of EGFP + phagocyte recruitment in the ear pinna tissue, and observations of bacterial injection sites showing a considerable recruitment of leukocytes for both bacterial forms. *S. aureus* is known to produce toxins and enzymes, such as leukocidins and coagulase, that are able to induce cell death by apoptosis, necroptosis, and also pyroptosis (Soe et al., 2021). This was coherent with ear pinna tissue necrosis observed as early as day 3. However, the exact proportion of each cell death pathway is yet to be studied.

We further determined the chemokine and cytokine profile in both cutaneous ear tissue and dLNs after *S. aureus* infection. In the ear tissue, we found large proportions of the PMN chemoattractant CXCL1 which has been shown previously to be notably secreted by keratinocytes in response to *S. aureus* (Olaru and Jensen, 2010). This would partially explain the significant increase in EGFP + immune cells observed at 24 h pi. High amounts of CXCL9/MIG, a Th1 cell-recruiting chemokine, were also found in infected mice. Globally, we found a robust pro-inflammatory cytokine response comparable between bacterial forms in both infected tissues, due to the presence of IL-1β, IL-6, and TNF-α. In line with these data, the presence of IL-17 may indicate that infected mice also mount a Th17 response, which is coherent with a previous study implicating IL-17 in establishing an effective antimicrobial response by γδ T cells (Cho et al., 2010). At early time points, IL-17 could also be produced by innate lymphoid cells in the skin and the dLNs (Cua and Tato, 2010). The absence of IFNγ production in infected mice could be explained by the early time points and/or by the presence of IL-10 in the infectious milieu, as the two are negatively correlated (Saraiva and O’Garra, 2010). Biofilms have indeed been shown to skew host immune responses toward an anti-inflammatory phenotype, in part *via* IL-10 secretion by M2 polarized MΦs and recruited MDSCs (Thurlow et al., 2011; Heim et al., 2018). In this study, the presence of IL-10 was comparable in both planktonic and biofilm infected ear tissues. In a previous study (Heim et al., 2020), *S. aureus* bacteria derived lactate was shown to inhibit HDAC11 in MΦs and MDSCs, which led to an increase in histone 3 acetylation at the IL-10 promoter, thus enhancing its expression. This discovery was, however, demonstrated in a biofilm prosthetic joint infection model, without comparison with planktonic bacteria. This anti-inflammatory response may be dependent on bacterial concentration, as IL-10 was not significantly increased in auricular dLNs of infected mice. Depending on the strain used, the inocula doses and the target tissues analyzed, some discrepancies may be observed between the inflammatory and anti-inflammatory chemokines and cytokines measured in different *in vivo* models. In all cases, host immune responses to *S. aureus* biofilm infection may be modulated by quorum sensing or metabolically dampened bacteria induced by host immune responses (Yamada and Kielian, 2018).

The use of intravital confocal imaging to further decorticate very early inflammatory responses allowed us to highlight differences in cellular behavior. First, we validated the previously observed “biofilm” phenotype that leukocytes present at the injection site, with cells arrested at the periphery of the biofilm and modified motility parameters of recruited cells (Abdul Hamid et al., 2020; Sauvat et al., 2020). This means that the protective barrier role that biofilms exert are common between these two *S. aureus* strains, and probably among all biofilm-producing strains. The two strains used in our previous works were both MSSAs, but a “biofilm” phenotype has also been described for the MRSA strain USA300 (Thurlow et al., 2011; Gries et al., 2020b). The use of biofilms grown over longer periods (3 or 4 days) in the latter experiments compared to those used in this report further suggest a common immune evasion mechanism conserved throughout biofilm maturation. Furthermore, the fate of phagocytes capable of penetrating *S. aureus* biofilms have been explored *in vitro*. For example, Bhattacharya et al. (2020) showed that biofilms provoked NET formation in penetrating PMNs *via* the activity of Panton-Valentine Leukocidins and γ-hemolysin AB. Otherwise, Thurlow et al. (2011) showed that MΦs closer to the base of biofilms were not viable, while those on the surface were intact. In the ear pinna model, the “biofilm” phenotype also extended to a difference in migration properties, namely average speed, displacement, and trajectory straightness between the two bacterial forms. Indeed, EGFP + phagocytes possessed decreased values for all three motility parameters stated previously in the presence of biofilms, meaning that cells moved at a slower rate, were more restrained and were not recruited directly toward biofilms. This could be partly explained by the inability of phagocytes to bind to bacterial components embedded in the extracellular matrix or by the expression of chemotaxis inhibitory protein of *Staphylococcus* (CHIPS) (de Vor et al., 2020). Observing bacteria-cell interactions using higher magnification objectives allowed us to show for the first time *in vivo* that phagocytes interacting with biofilms were less capable of emitting pseudopods (illustrated by the decrease in cell perimeter) in the first hours after bacteria inoculation. Previously, Jesaitis et al. (2003) have shown a comparable phenomenon *in vitro* with PMNs and *Pseudomonas aeruginosa* biofilms. In these experiments, PMNs were immobilized on the surface of biofilms, while conserving a rounded morphology, a characteristic of unstimulated phagocytes. Unfortunately, the dynamics of EGFP^+^ cells could not be further analyzed by intravital imaging due to ear tissue swelling and necrosis starting at 24 h pi. In line with these results, we found that the expression of the activation marker CD11b in PMNs (Kinhult et al., 2003) of biofilm-infected mice was similar to that of controls at 2 h pi, while it was upregulated in cells in the presence of planktonic bacteria.

Complementing these findings with FACS analysis allowed us to quantify the proportion of phagocytic cells recruited to both ear and dLN tissue over the first few days, and also to evaluate phagocytosis efficacy of recruited cells. Our results show that the majority of EGFP + cells that were observed in the cutaneous injection sites as early as 2 h pi were PMNs. After 24 h and onward, PMNs comprised the main bulk of the myeloid cell response, suggesting an important role played by these cells in the *S. aureus* early immune responses. Interestingly, at 2 h pi, increased PMN numbers were only observed in response to biofilms alluding to biofilm specific properties that favor PMN recruitment. In the Gram-negative bacterium *Pseudomonas aeruginosa*, the quorum sensing molecule N-acylhomoserine lactone acted as a strong PMN chemoattractant (Holm and Vikström, 2014). Recruitment continued at 24 and 48 h pi, coherent with the high levels of CXCL1 found in ear skin tissue at these time points.

In the dLN, kinetics of PMN recruitment were mostly similar to those observed in the ear tissue, except for a significant difference between both infected conditions in PMN percentage after 48 h. The recruitment of PMNs over the course of time was coherent with the large quantity of CXCL1 found in the skin and the dLN. As almost the same number of cells could be observed in both tissues despite the gap in CXCL1 concentration observed in the dLN, this suggests the implication of other homing mechanisms toward dLN. Moreover, the decrease in PMN numbers in the dLN of biofilm infected mice at 48 h pi, as compared to the planktonic condition, seemed more pronounced than in the ear tissue.

MO/MΦ arrived at the injection site after PMNs, but only after 48 h, and always in a smaller proportion compared to PMNs. Indeed, an initial increase in MO/MΦ numbers in biofilm mice compared to planktonic mice was observed at 2 h pi, but the two were however comparable to controls. Moreover, MO/MΦ were also recruited in control mice between 2 and 24 h pi, which could be reflective of their non-specific recruitment after skin trauma due to intradermal injection of PBS.

The numbers of recruited MO/MΦ were almost comparable to those of PMNs in the dLN, which coincides with the large quantity of CXCL9 predominant at 24 h pi. This chemokine has been previously shown to induce MΦ migration and activation in a apical periodontitis mouse model (Hasegawa et al., 2021). Resch et al. (2005) described the upregulation of polysaccharide intercellular adhesins, staphylococcal secretory antigen and staphyloxanthin during biofilm formation, as compared to planktonic growth. The recruited MOs/MΦs are antigen presenting cells that will migrate to the cutaneous dLN. Their mobilization from the skin and probably also from the blood circulation is clearly detected in the auricular dLN from 24 h pi. They will present antigens to naïve T cells, therefore playing a key role in inducing specific adaptive immune responses to biofilms. These responses need to be further explored.

In further experiments, the functional properties of PMNs recruited at the cutaneous injection sites were analyzed, as they were the major player in inflammatory responses mounted against *S. aureus* in this model.

CD11b is a β2 integrin that is found on the cell surface and also PMN granules. Following activation, intracellular pools of CD11b are translocated to the cell surface where they form heterodimers with CD18 capable of interacting with fibrinogen and inactivated complement component 3 (iC3b) (Solovjov et al., 2005). The latter is implicated in opsonization, thus increasing phagocytosis effectiveness in PMNs with higher levels of CD11b. PMNs in contact with biofilms did not overexpress the CD11b surface marker at the beginning of the infection period. This could be due to the physical barrier represented by biofilms which could limit PMN access to bacteria. However, higher levels of CD11b were detected 48 h pi despite the PMNs decreased numbers observed at this later time point. This difference between the two inocula after 48 h could be due to the presence of matrix components in the biofilm inoculum, representing a multiplicity of additional antigens that are absent in the planktonic inoculum. Furthermore, a proportion of biofilm bacteria could be lysed after phagocytosis and matrix components could be released at the cutaneous injection sites. Namely, Phenol Soluble Modulins are biofilm matrix proteins well known as immunomodulators (Richardson et al., 2018). They also have been shown to fix to PMN formyl peptide receptors inducing their recruitment. The activation of this PMN receptor has been shown to induce chemotaxis, ROS production, degranulation, cytokine expression, and phagocytosis (Cheung et al., 2014; Dorward et al., 2015).

Analysis of bacterial association to immune cells showed that PMNs were the predominant immune cell interacting with inoculated *S. aureus* throughout the first few days of infection. Inversely, MO/MΦ were less likely to interact with bacteria, as they were detected in lesser proportions (number and percentage) compared to PMNs at each time-point. In just 2 h, PMNs present at the injection site were able to interact with planktonic and biofilm bacteria, corroborating intravital imaging observations. In response to large aggregates of bacteria and phagocytosis failure, PMNs release NETs composed of chromatin and antimicrobial peptides which subsequently trap and degrade bacteria (Branzk et al., 2014). Furthermore, this process causes cell membrane rupture and thus cell death although certain studies have shown that some PMNs conserve membrane integrity after NET release (non-lytic NETosis) and continue to phagocytose bacteria (Bhattacharya et al., 2020). The proportion of cells undergoing this type of NETosis in the ear pinna model is yet to be examined.

Phagocytic capacity of recruited PMNs was further studied. Phagocytosis of biofilm bacteria was increased at 48 h pi, as compared to planktonic bacteria, while it was comparable for both bacterial forms 24 h pi. In line with these results, a higher proportion of PMNs containing bacterial aggregates of 5 or more at 24 h pi was also observed in MO/MΦ. This could be explained by the spatial distribution of bacteria that are more compact in biofilms, allowing greater entry into cells during phagocytosis. Planktonic and biofilm bacteria that were observed intracellularly (in PMNs and MO/MΦ) were shown to be viable, and at comparable levels between the two forms. This proves the *in vivo* ability of *S. aureus* to maintain intracellularly inside phagocytes. Indeed, to combat degradation, *S. aureus* produce a variety of enzymes ranging from extracellular adherence protein that inhibit PMN serine protease (Stapels et al., 2014) to catalase and superoxide dismutase that prevent the oxidation effects of ROS (Painter et al., 2015). However, the exact *in vivo* contribution of persisting bacteria or of new phagocytosed bacteria to the proportion of detected intracellular bacteria in the ear tissue is not known.

A previous study suggested that PMN accumulation in *S. aureus* infected skin tissue was attributed to blood stream recruitment of mature PMNs, prolonged survival of recruited PMNs and local proliferation/maturation of c-kit^+^ progenitor cells into mature PMNs (Kim et al., 2011). These progenitor cells were also capable of differentiating into MDSCs. The latter were initially described as having an important role in cancer where they promote an immunosuppressive microenvironment *via* the secretion of IL-10 and TGFβ or high levels of ROS, which have been associated with T-cell deactivation. They also have been identified in *S. aureus* biofilm prosthetic joint infections where they contribute toward anti-inflammatory responses (Heim et al., 2014). In the described mouse models, MDSCs were identified based on the co-expression of surface markers CD11b, Ly6G, and Ly6C. The selection of our PMN population was based on the same markers. In this report, even if PMN numbers were decreased after 48 h of biofilm infection as compared to the planktonic condition, these cells globally expressed higher levels of CD11b, had an increased production of ROS, and an increased phagocytic capacity. These results illustrate a potential PMN “over-activated” status in response to biofilms after 48 h. Further experiments are required to identify the specific phenotype of the different PMN subsets induced by *S. aureus* biofilms at this time point and their possible MDSC nature.

Taken globally, at early time points (2 h), we observed an initial increase in PMN numbers associated with CD11b expression levels comparable to controls, and a decreased capacity to penetrate biofilms and to emit pseudopods. At later time points (48 h), we observed a decrease in PMN numbers with a potential “over-activated” status associated with increased CD11b expression levels, ROS production and phagocytic capacity. In these conditions, we could have expected a higher and lower biofilm bacterial load after 2 and 48 h, respectively. Surprisingly, no differences in CFU numbers were observed with the planktonic condition in all experiments performed, suggesting a more complex mechanism of bacterial survival at play involving other immune or non-immune cell populations. Namely, Natural Killer cells that are recruited to *S. aureus* skin infections have been reported to secrete IL-17, subsequently activating keratinocytes to produce pro-inflammatory cytokines, chemokines and adhesion molecules that mediate neutrophil recruitment (Krishna and Miller, 2012). In any case, this comparative study where immune responses and bacterial loads were followed over time has never been carried out, and thus gives new insights into the understanding of innate immune responses directed toward *S. aureus* biofilms.

In conclusion, the ear pinna model allowed us to obtain a global picture of the early dynamics of immune responses against both forms of *S. aureus* at different levels of the organism. By performing a direct comparison of innate immune responses between planktonic and biofilm bacteria in the cutaneous tissue and dLN, we provide evidence that biofilms elicit specific immune signatures regarding PMN kinetics of recruitment and functional properties at very early time points.

## Data Availability Statement

The raw data supporting the conclusions of this article will be made available by the authors, without undue reservation.

## Ethics Statement

The animal study was reviewed and approved by the Ethics Committee on Animal Experimentation (Auvergne C2E2A, Clermont-Ferrand, France) Agreement number: 1,725.

## Author Contributions

AA, AC, AD, JJ, FL, and PG conceived and designed the experiments. AA analyzed the data. AA, AC, AD, and PG performed experiments. AA, AC, AD, JJ, and PG participated in the provision of materials for experiments. PG supervised, managed, and coordinated responsibility for the research activity. AA and PG verified the overall replication and reproducibility of results, visualized, and wrote the manuscript. All authors contributed to the article and approved the submitted version.

## Conflict of Interest

The authors declare that the research was conducted in the absence of any commercial or financial relationships that could be construed as a potential conflict of interest.

## Publisher’s Note

All claims expressed in this article are solely those of the authors and do not necessarily represent those of their affiliated organizations, or those of the publisher, the editors and the reviewers. Any product that may be evaluated in this article, or claim that may be made by its manufacturer, is not guaranteed or endorsed by the publisher.

## Supplementary Material

Supplementary Figure 1 Representative images illustrating the different levels of scoring used to assess ear pinna tissue inflammation. Images of C57BL/6J mice ear pinnae showing erythema (black empty arrowheads) at different time points following infection by *S. aureus*.

Supplementary Figure 2 Mosaic acquisition of LysM-EGFP transgenic mice ear pinna tissue. Reconstituted confocal images of LysM-EGFP transgenic mice ear pinna tissue following micro-injection of PBS **(A)** or mCherry-SH1000 planktonic bacteria **(B)**. Images correspond to the maximal projection intensities of the EGFP signal, and the yellow line indicates the ROI where the “Sum of EGFP fluorescence intensities” was measured. Scale bar: 2 mm. One representative experiment is shown for each group of mice from at least 3 independent experiments.

Supplementary Figure 3 Cytokine production in the ear pinna tissue and dLNs of control and planktonic or biofilm infected mice. **(A–H)** Cytokine levels, expressed in pg/mg of total protein, were analyzed by Bioplex in the supernatants of ear pinna tissue **(A,C,E,G)** and dLN **(B,D,F,H)** homogenates at 24 and 72 h pi (median ± IQR, number of mice: N_*C*_ = 5, N_*P/BF*_ = 6, from at least 3 different experiments, Mann-Whitney two-tailed test, ^∗^*p* < 0.05).

Supplementary Figure 4 Representative images of EGFP + immune cell recruitment to either *S. aureus* planktonic or biofilm bacteria. **(A–D)** Live confocal imaging, using X10 objectives, in the ear pinna tissue of LysM-EGFP transgenic mice following micro-injection of mCherry-SH1000 planktonic **(A,B)** or biofilm **(C,D)** bacteria. Representative average projections of green (EGFP + innate immune cells) and magenta (bacteria) fluorescence, acquired at 2.10 h pi **(A)**, 3.30 h pi **(B)**, 2.10 h pi **(C)**, and 3.30 h pi **(D)**, show immune cells recruited toward the injection site. Asterisk: autofluorescent hair (also in magenta). Scale bar: 100 μm. **(E–H)** Live confocal imaging, using X40 objectives, in the ear pinna tissue of LysM-EGFP transgenic mice following micro-injection of mCherry-SH1000 planktonic **(E,F)** or biofilm **(G,H)** bacteria. Maximum projections of green (innate immune cells) and magenta (bacteria) fluorescence, acquired at 4.20 h pi **(E)**, 3.25 h pi **(F)**, 3.25 h pi **(G)**, and 2.30 h pi **(H)** show immune cells in the injection area. The yellow line indicates the ROI where cell perimeter was measured. Asterisk: autofluorescent hair (also in magenta). Scale bar: 15 μm. One representative experiment is shown for each group of mice from at least 9 independent experiments.

Supplementary Figure 5 Flow cytometry gating strategy for myeloid cells populations isolated from the ear tissue and dLN. Representative dot plots showing the gating strategy to analyze myeloid cell populations in the skin and dLN following inoculation. Representative dot plots and percentages of cells gated are shown from planktonic infected WT C57BL/6J mice at 24 h pi.

Supplementary Figure 6 Immune cell counts and percentage of bacteria associated immune cells. **(A,B)** Total number of live cells recruited to ear pinna tissue **(A)** and dLNs **(B)** of control and planktonic or biofilm infected mice from 2 h pi to day 2 pi (median ± IQR, number of mice: N_*C*_ = 12–15, N_*P*_ = 9–12, N_*BF*_ = 9–15, from at least 3 different experiments, Mann-Whitney two-tailed test, ^∗^*p* < 0.05). **(C,D)** GFP median fluorescence intensity of PMNs **(C)** and MOs/MΦs **(D)** associated to GFP-SH1000 in the ear pinna tissues of control and planktonic or biofilm infected mice from 2 to 48 h pi (median ± IQR, number of mice: N_*C*_ = 12–15, N_*P*_ = 9–12, N_*BF*_ = 9–15, from at least 3 different experiments, Mann-Whitney one-tailed test, ^∗^*p* < 0.05). **(E,F)** Percentage of bacteria associated PMNs **(E)** and MOs/MΦs **(F)** in ear pinna tissue of control and planktonic or biofilm infected mice from 2 h pi to day 2 pi (median ± IQR, number of mice: N_*C*_ = 12–15, N_*P*_ = 9–12, N_*BF*_ = 9–15, from at least 3 different experiments, Mann-Whitney two-tailed test, ^∗^*p* < 0.05).

Supplementary Figure 7 Phagocytosis of *S. aureus* planktonic or biofilm bacteria by CD11b^+^ myeloid cells in the ear pinna tissue. **(A,B)** Transmitted light images of CD11b^+^ cells, following isolation from ear pinna tissue onto glass slides and MGG staining, show PMNs **(A,B)** and MOs/MΦs **(B)** harboring intracellular bacteria (black arrowheads). Representative images are shown from planktonic infected WT C57BL/6J mice at 24 h pi **(A)** and 48 h pi **(B)**. **(C)** Percentage of MOs/MΦs containing intracellular bacteria assessed at 24 and 48 h pi (mean ± SEM, number of cells: N_*P*_ = 223–238, N_*BF*_ = 232–338, from at least 3 different experiments, Mann-Whitney one-tailed test, ^∗^*p* < 0.05). **(D)** Percentage of MOs/MΦs containing 0, 1–2, 3–4, or 5 or more intracellular bacteria assessed at 24 and 48 h pi (mean ± SEM, number of cells: N_*P*_ = 223–238, N_*BF*_ = 232–338, from at least 3 different experiments, Mann-Whitney one-tailed test, ^∗^*p* < 0.05). **(E)** Quantification of intracellular bacteria, expressed in CFUs/10^6^ cells, in CD11b + cells isolated from ear pinna tissue of planktonic or biofilm infected mice, at 24 and 48 h pi (median ± IQR, number of mice: N_*P*_ = 5, N_*BF*_ = 3–4, from at least 3 different experiments, Mann-Whitney one-tailed test, ^∗^*p* < 0.05).

Supplementary Movie 1 EGFP + innate immune cells are recruited toward a planktonic injection site. *In vivo* confocal time-lapse imaging of immune cell migration in LysM-EGFP transgenic mice ear tissue injected with planktonic bacteria from 2.50 to 3.10 hpi. Average projections of time-lapse images. Z-stacks collected 72.98 s apart. Scale bar: 100 μm.

Supplementary Movie 2 EGFP + innate immune cells are recruited toward a biofilm injection site. *In vivo* confocal time-lapse imaging of immune cell migration in LysM-EGFP transgenic mice ear tissue injected with planktonic bacteria from 2.55 to 3.25 hpi. Average projections of time-lapse images. Z-stacks collected 106.55 s apart. Scale bar: 100 μm.
