# Supplementary figures and images for: Differential Early in vivo Dynamics and Functionality of Recruited Polymorphonuclear Neutrophils After Infection by Planktonic or Biofilm Staphylococcus aureus
Source: Front Microbiol. 2021 Aug 30;12:728429. doi: 10.3389/fmicb.2021.728429 (PMC8435793; doi:10.3389/fmicb.2021.728429)

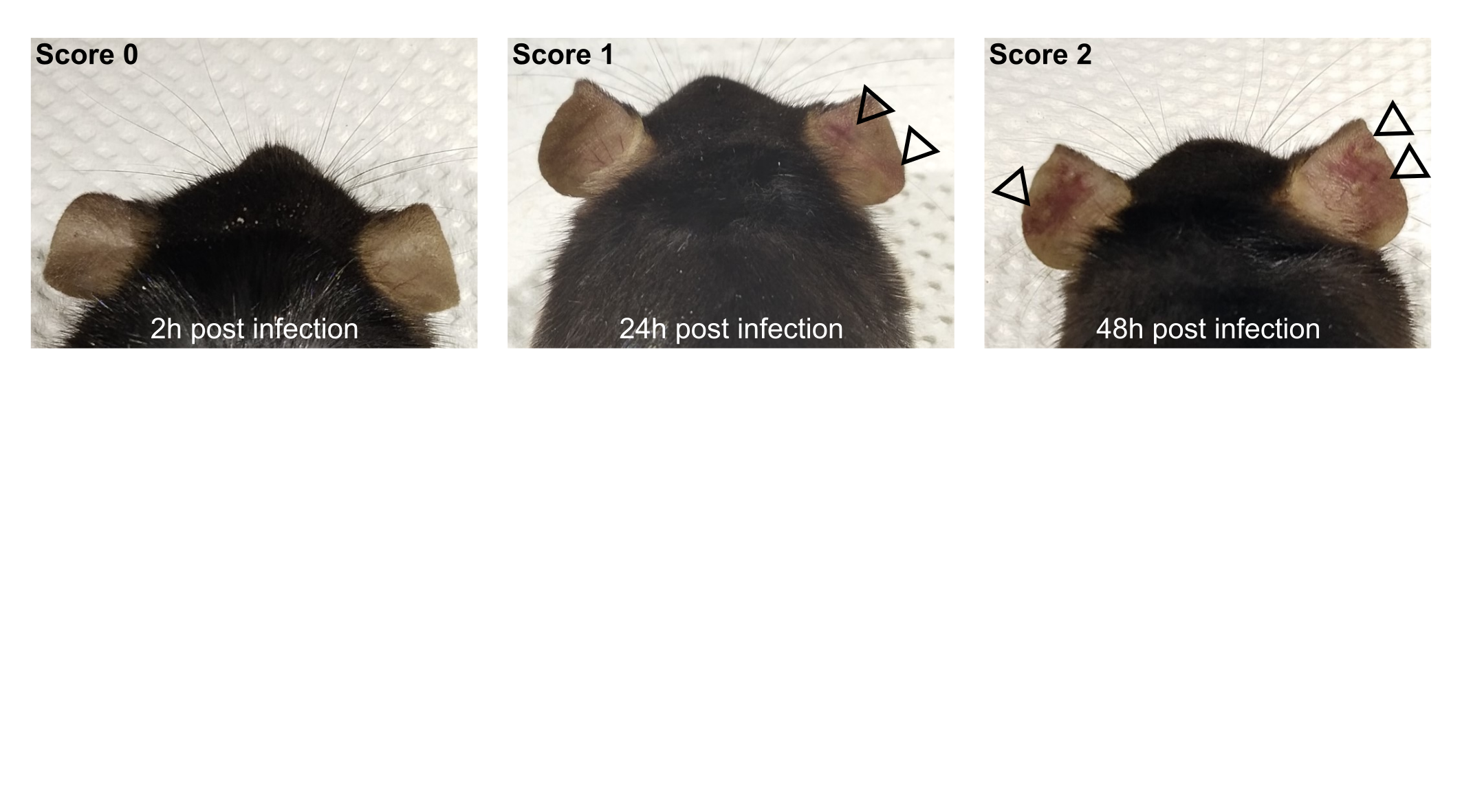

Supplement: Supplementary Figure 1 — Representative images illustrating the different levels of scoring used to assess ear pinna tissue inflammation. Images of C57BL/6J mice ear pinnae showing erythema (black empty arrowheads) at different time points following infection by S. aureus. [file Image_1.TIF]

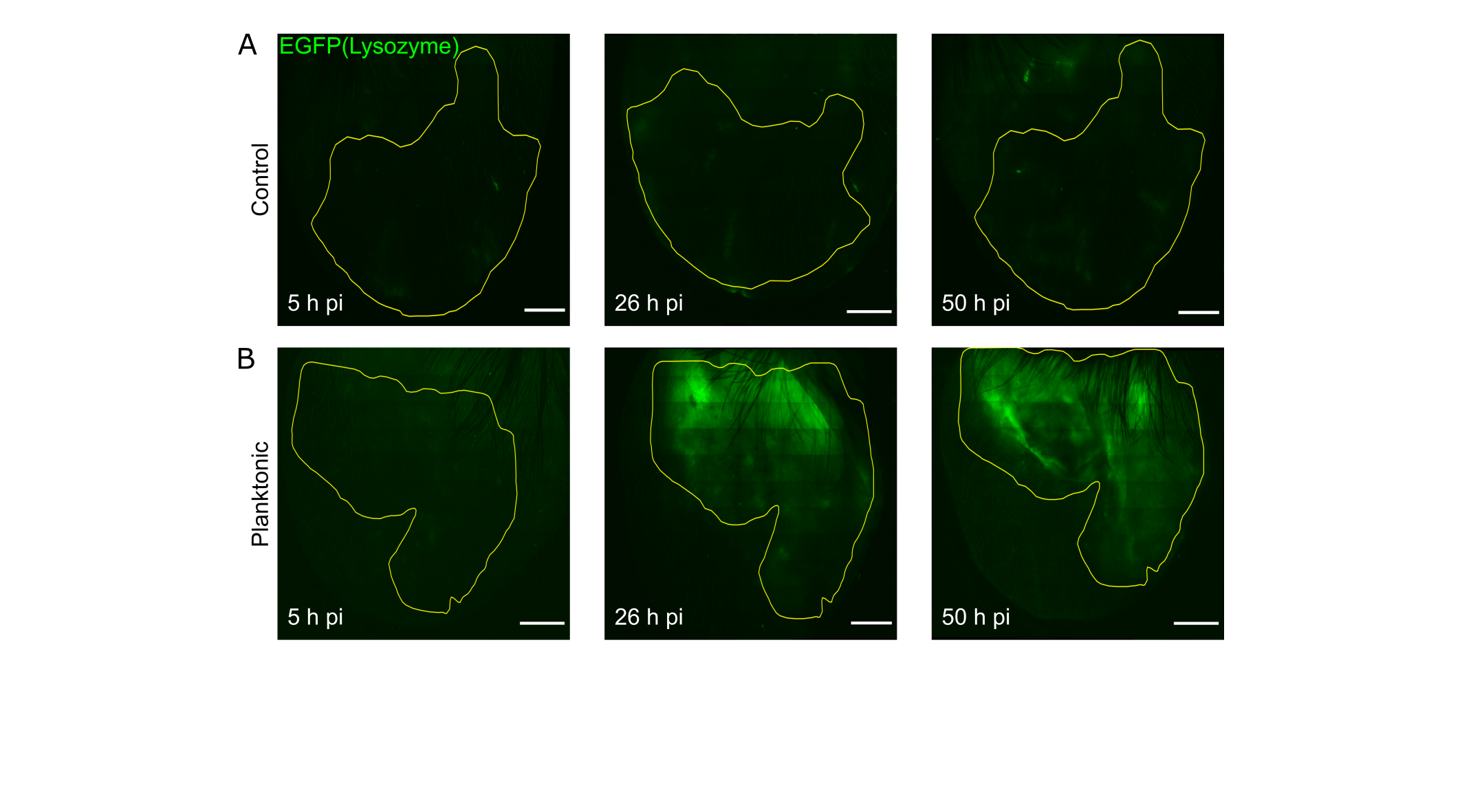

Supplement: Supplementary Figure 2 — Mosaic acquisition of LysM-EGFP transgenic mice ear pinna tissue. Reconstituted confocal images of LysM-EGFP transgenic mice ear pinna tissue following micro-injection of PBS (A) or mCherry-SH1000 planktonic bacteria (B). Images correspond to the maximal projection intensities of the EGFP signal, and the yellow line indicates the ROI where the “Sum of EGFP fluorescence intensities” was measured. Scale bar: 2 mm. One representative experiment is shown for each group of mice from at least 3 independent experiments. [file Image_2.TIF]

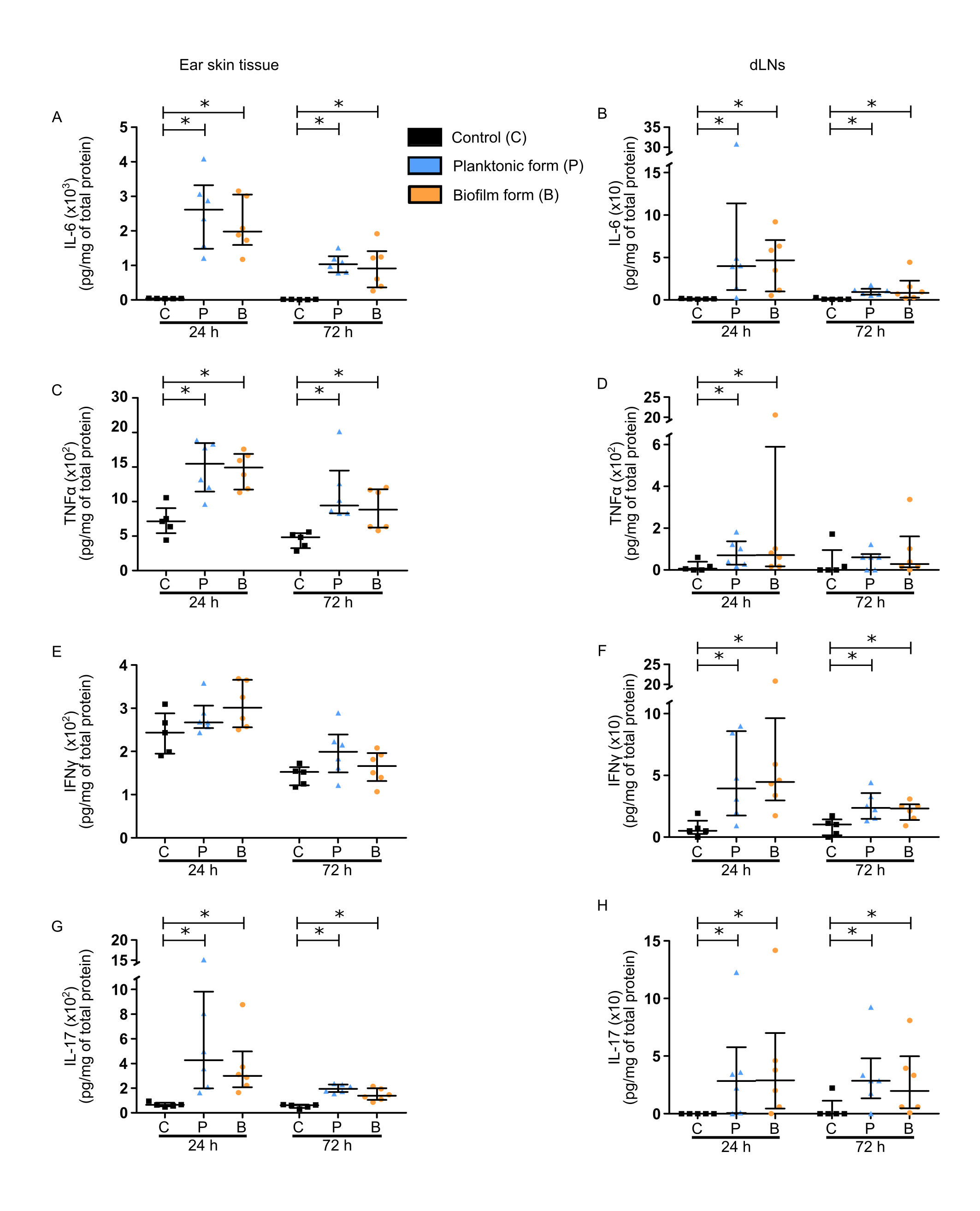

Supplement: Supplementary Figure 3 — Cytokine production in the ear pinna tissue and dLNs of control and planktonic or biofilm infected mice. (A–H) Cytokine levels, expressed in pg/mg of total protein, were analyzed by Bioplex in the supernatants of ear pinna tissue (A,C,E,G) and dLN (B,D,F,H) homogenates at 24 and 72 h pi (median ± IQR, number of mice: NC = 5, NP/BF = 6, from at least 3 different experiments, Mann-Whitney two-tailed test, ∗p < 0.05). [file Image_3.TIF]

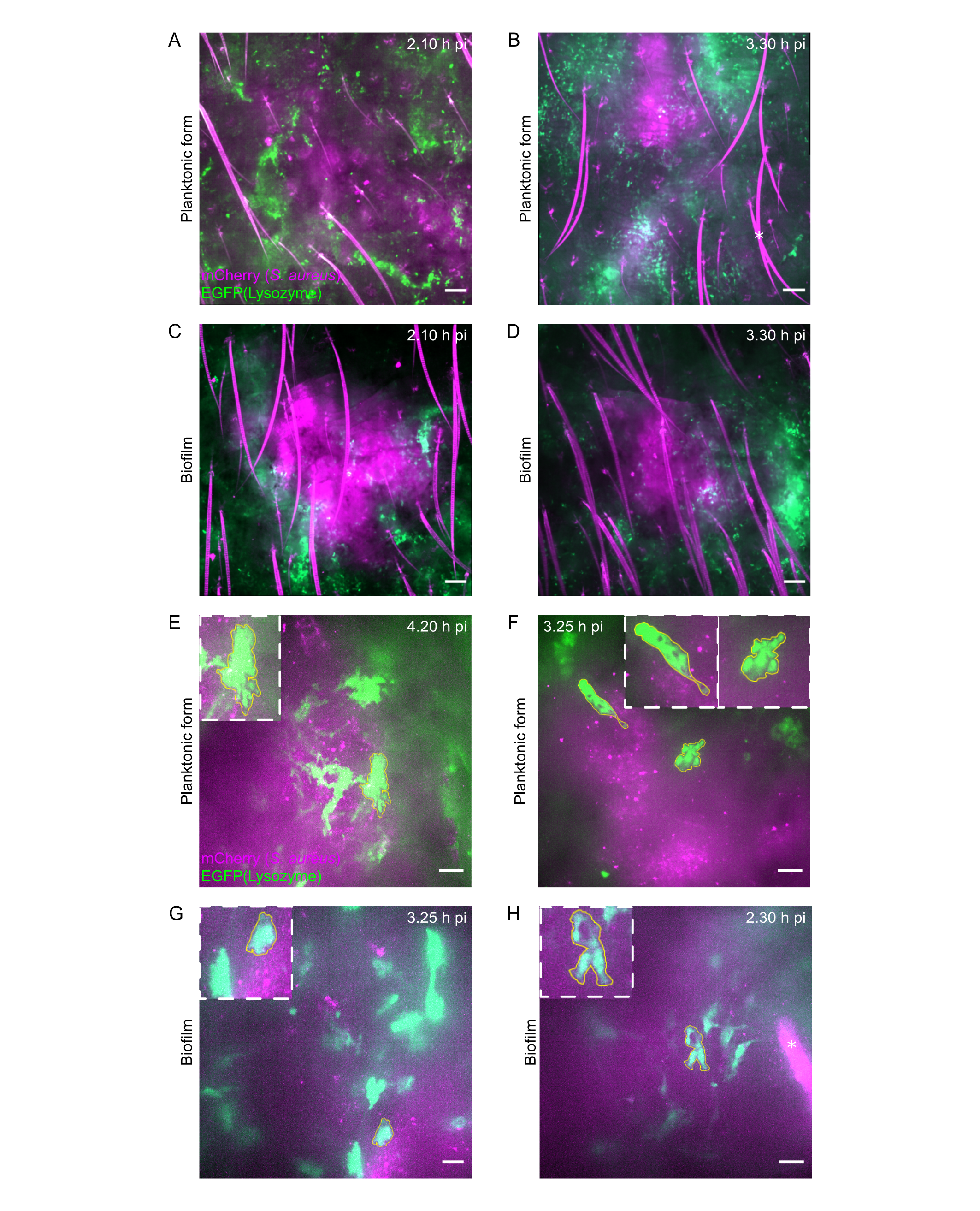

Supplement: Supplementary Figure 4 — Representative images of EGFP + immune cell recruitment to either S. aureus planktonic or biofilm bacteria. (A–D) Live confocal imaging, using X10 objectives, in the ear pinna tissue of LysM-EGFP transgenic mice following micro-injection of mCherry-SH1000 planktonic (A,B) or biofilm (C,D) bacteria. Representative average projections of green (EGFP + innate immune cells) and magenta (bacteria) fluorescence, acquired at 2.10 h pi (A), 3.30 h pi (B), 2.10 h pi (C), and 3.30 h pi (D), show immune cells recruited toward the injection site. Asterisk: autofluorescent hair (also in magenta). Scale bar: 100 μm. (E–H) Live confocal imaging, using X40 objectives, in the ear pinna tissue of LysM-EGFP transgenic mice following micro-injection of mCherry-SH1000 planktonic (E,F) or biofilm (G,H) bacteria. Maximum projections of green (innate immune cells) and magenta (bacteria) fluorescence, acquired at 4.20 h pi (E), 3.25 h pi (F), 3.25 h pi (G), and 2.30 h pi (H) show immune cells in the injection area. The yellow line indicates the ROI where cell perimeter was measured. Asterisk: autofluorescent hair (also in magenta). Scale bar: 15 μm. One representative experiment is shown for each group of mice from at least 9 independent experiments. [file Image_4.TIF]

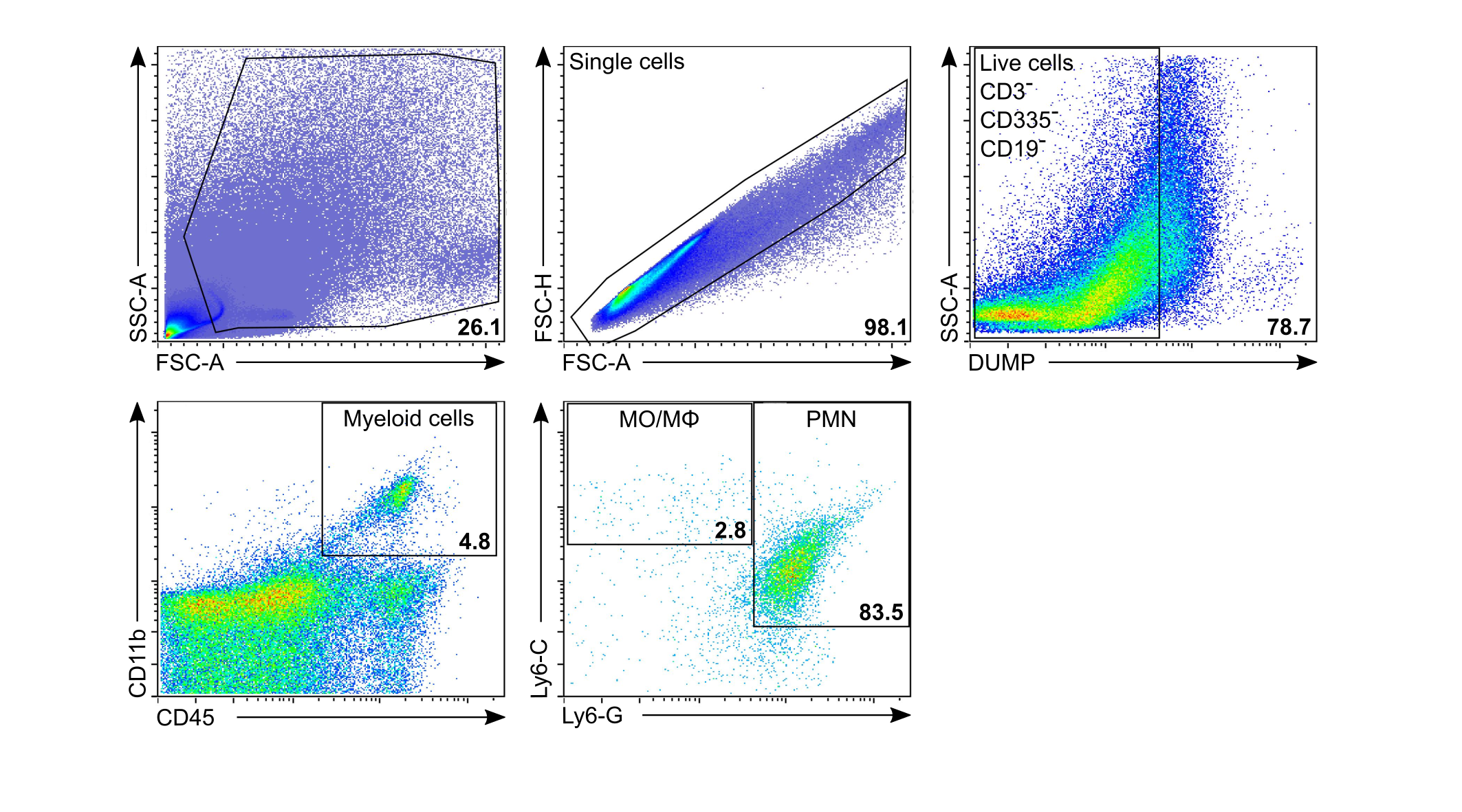

Supplement: Supplementary Figure 5 — Flow cytometry gating strategy for myeloid cells populations isolated from the ear tissue and dLN. Representative dot plots showing the gating strategy to analyze myeloid cell populations in the skin and dLN following inoculation. Representative dot plots and percentages of cells gated are shown from planktonic infected WT C57BL/6J mice at 24 h pi. [file Image_5.TIF]

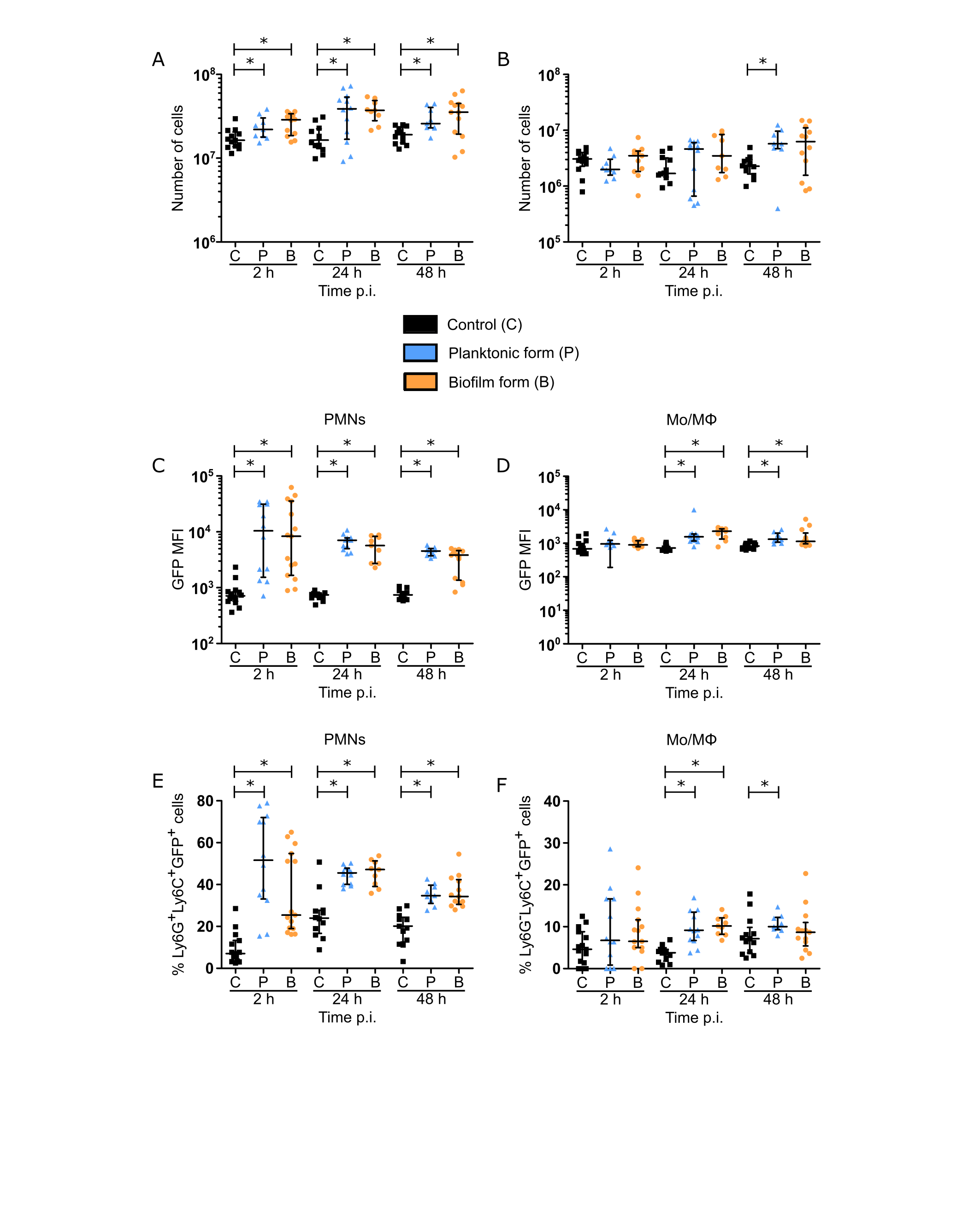

Supplement: Supplementary Figure 6 — Immune cell counts and percentage of bacteria associated immune cells. (A,B) Total number of live cells recruited to ear pinna tissue (A) and dLNs (B) of control and planktonic or biofilm infected mice from 2 h pi to day 2 pi (median ± IQR, number of mice: NC = 12–15, NP = 9–12, NBF = 9–15, from at least 3 different experiments, Mann-Whitney two-tailed test, ∗p < 0.05). (C,D) GFP median fluorescence intensity of PMNs (C) and MOs/MΦs (D) associated to GFP-SH1000 in the ear pinna tissues of control and planktonic or biofilm infected mice from 2 to 48 h pi (median ± IQR, number of mice: NC = 12–15, NP = 9–12, NBF = 9–15, from at least 3 different experiments, Mann-Whitney one-tailed test, ∗p < 0.05). (E,F) Percentage of bacteria associated PMNs (E) and MOs/MΦs (F) in ear pinna tissue of control and planktonic or biofilm infected mice from 2 h pi to day 2 pi (median ± IQR, number of mice: NC = 12–15, NP = 9–12, NBF = 9–15, from at least 3 different experiments, Mann-Whitney two-tailed test, ∗p < 0.05). [file Image_6.TIF]

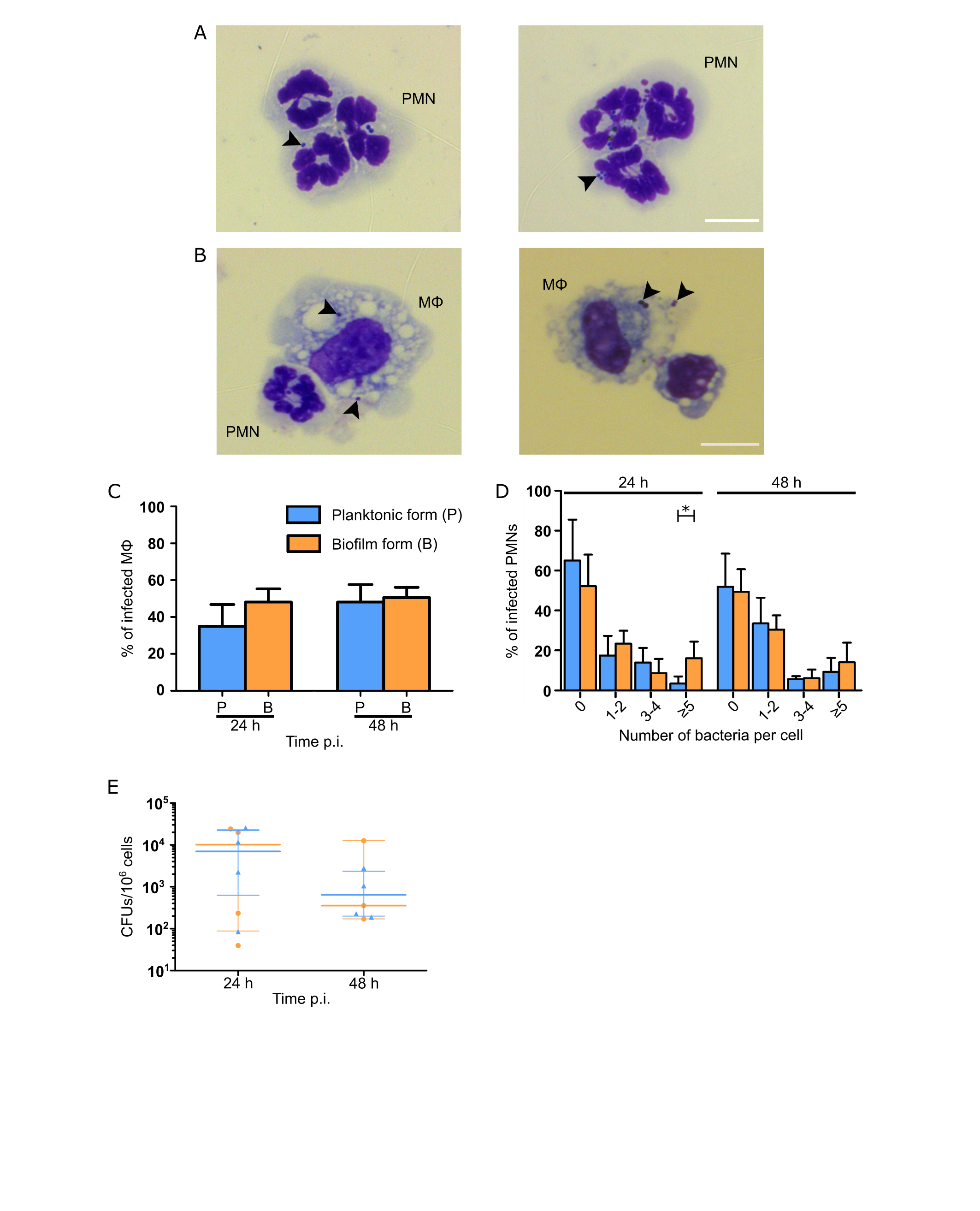

Supplement: Supplementary Figure 7 — Phagocytosis of S. aureus planktonic or biofilm bacteria by CD11b+ myeloid cells in the ear pinna tissue. (A,B) Transmitted light images of CD11b+ cells, following isolation from ear pinna tissue onto glass slides and MGG staining, show PMNs (A,B) and MOs/MΦs (B) harboring intracellular bacteria (black arrowheads). Representative images are shown from planktonic infected WT C57BL/6J mice at 24 h pi (A) and 48 h pi (B). (C) Percentage of MOs/MΦs containing intracellular bacteria assessed at 24 and 48 h pi (mean ± SEM, number of cells: NP = 223–238, NBF = 232–338, from at least 3 different experiments, Mann-Whitney one-tailed test, ∗p < 0.05). (D) Percentage of MOs/MΦs containing 0, 1–2, 3–4, or 5 or more intracellular bacteria assessed at 24 and 48 h pi (mean ± SEM, number of cells: NP = 223–238, NBF = 232–338, from at least 3 different experiments, Mann-Whitney one-tailed test, ∗p < 0.05). (E) Quantification of intracellular bacteria, expressed in CFUs/106 cells, in CD11b + cells isolated from ear pinna tissue of planktonic or biofilm infected mice, at 24 and 48 h pi (median ± IQR, number of mice: NP = 5, NBF = 3–4, from at least 3 different experiments, Mann-Whitney one-tailed test, ∗p < 0.05). [file Image_7.tif]
